# Differential Responses of Arctic Vegetation to Nutrient Enrichment by Plankton- and Fish-Eating Colonial Seabirds in Spitsbergen

**DOI:** 10.3389/fpls.2016.01959

**Published:** 2016-12-27

**Authors:** Adrian Zwolicki, Katarzyna Zmudczyńska-Skarbek, Jan Matuła, Bronisław Wojtuń, Lech Stempniewicz

**Affiliations:** ^1^Department of Vertebrate Ecology and Zoology, University of GdańskGdańsk, Poland; ^2^Institute of Biology, The Faculty of Biology and Animal Science, Wrocław University of Environmental and Life SciencesWrocław, Poland; ^3^Department of Botany and Plant Ecology, The Faculty of Life Sciences and Technology, Wrocław University of Environmental and Life SciencesWrocław, Poland; ^4^Department of Ecology, Biogeochemistry and Environmental Protection, Faculty of Biological Sciences, University of WrocławWrocław, Poland

**Keywords:** guano deposition, bird cliff vegetation, plant communities, soil chemistry, little auk, kittiwake, guillemot

## Abstract

The role of seabirds as sea-land biovectors of nutrients is well documented. However, no studies have examined whether and how colonial seabirds that differ in diet may influence terrestrial vegetation. Therefore, the purpose of the study was to describe and compare plant communities located in the vicinity of the two most common types of seabird colonies in Arctic, occupied by piscivorous or planktivorous species. Within 46 plots arranged in four transects in the vicinity of planktivorous (little auk, *Alle alle*) and piscivorous colonies (mixed colony of Brunnich’s guillemot, *Uria lomvia*, and black-legged kittiwake, *Rissa tridactyla*) we measured the following: guano deposition, physical and chemical characteristics of soil, total nitrogen and its stable isotope signatures in soil and plants, ground vegetation cover of vascular plants and mosses, and the occurrence of lichens, algae and cyanobacteria. Using LINKTREE analysis, we distinguished five plant communities, which reflected declining influence along a birds fertilization gradient measured as guano deposition. SIMPROOF test revealed that these communities differed significantly in species composition, with the differences related to total soil nitrogen content and δ^15^N, distinctive levels of phosphates, potassium and nitrates, and physical soil properties, i.e., pH, conductivity and moisture. The communities were also clearly distinguished by distance from the bird colony. The two colony types promoted development of specific plant communities: the immediate vicinity of the planktivorous colony characterized by a *Deschampsia alpina*–*Cerastium arcticum* community while under the piscivorous colony a *Cochlearia groenlandica–Poa alpina* community was present. Despite the similar size of the colonies and similar magnitude of guano input, differences between ornithogenic communities were connected mostly to phosphate content in the soil. Our results show that the guano input from seabirds which have different diets can affect High Arctic vegetation in specific and more complex ways than previously realized.

## Introduction

The chronic lack of nutrients and harsh climatic conditions of the polar regions result in terrestrial ecosystems generally having a simplified structure, with low primary production, and low species diversity (Odum, 1993). In this environment, seabirds acting as bio-vectors play a central role in tundra vegetation development (e.g., Wojciechowska et al., 2015; Zwolicki et al., 2016). They are intimately connected to the marine environment where they feed, while on land they form colonies in the breeding season as well as molt. Seabirds are among the most numerous birds in the world (del Hoyo et al., 1996). In the polar regions, they dominate the avifauna both in terms of the number of species and abundance. In the Arctic, the largest colonies are formed by a few common seabird species from the auk (Alcidae) and gull (Laridae) families, with the most numerous being the little auk, *Alle alle*. Its population is estimated at ca. 35 million pairs, making it the most abundant bird species from either polar region (Wojczulanis-Jakubas et al., 2011). Slightly less numerous are guillemots (Brünnich’s guillemot, *Uria lomvia*, and common guillemot, *U. aalge*) and the black-legged kittiwake, *Rissa tridactyla*, which often nests together with them, whose population numbers are estimated at 30, 14, and 18 million individuals, respectively (del Hoyo et al., 1996).

During the roughly 3 months of the polar breeding period seabirds deposit substantial amounts of marine derived organic matter on land (Bokhorst et al., 2007; Zwolicki et al., 2013, 2016). This consists primarily of excrement (guano) produced by chicks and adults, but also feathers, food remains, eggs and dead birds, all contributing to the nutrients available locally in the soil (Polis et al., 1997; Smith and Froneman, 2008). For instance, during one breeding season in Hornsund (south-west Spitsbergen), little auks provide ca. 60 t dry mass of feces km^-2^ within the breeding colony, and ∼25 t km^-2^ area around the colony (Stempniewicz, 1990). Such enormous amounts of fertilization, constituting locally the major source of nutrients for terrestrial ecosystems, have large impacts on arctic plant communities (Erskine et al., 1998; Ellis, 2005; Ellis et al., 2011; Zwolicki et al., 2015, 2016). Seabird nesting sites and their vicinities are characterized by much higher concentrations of ammonium, nitrate, phosphate, and many other salts as compared to areas beyond the influence of the birds (Anderson and Polis, 1999; García et al., 2002; Zwolicki et al., 2013, 2015). Local soil fertilization causes a significant increase in plant biomass and changes in life history strategy (Anderson and Polis, 1999; Vidal et al., 2003; Ellis et al., 2006; Zmudczyńska-Skarbek et al., 2013; Wojciechowska et al., 2015). The enhanced primary production in the vicinity of seabird colonies generates further changes in local trophic networks through encouraging greater use of ornithogenic tundra by herbivores (Jakubas et al., 2008). Accumulating organic matter consequently increases food resources for saprophytes, such as springtails and mites, which causes changes in community composition in these areas (Byzova et al., 1995; Zmudczyńska et al., 2012; Zawierucha et al., 2015; Zmudczyńska-Skarbek et al., 2015).

Hutchinson (1950) reported that the strong seabird influence on the habitat around their nesting sites resulted from several factors, including the high density of nests, feces usage as a nest-building material, and the deposition of a large amount of guano in the nest and around it. More recent studies have suggested that the response of the tundra ecosystem to marine-origin nutrient supplies may also be influenced by the birds’ diet (Stempniewicz et al., 2007; Mulder et al., 2011; Zwolicki et al., 2013). Such differential responses may be driven by differences in the chemical composition of the feces of birds that feed on plankton, fish or molluscs (Bédard et al., 1980). Furthermore, the microbial communities developing on guano produced by seabirds with different diets, and the amount and composition of products of their biochemical activities, may also vary (Stempniewicz et al., 2007).

Various phytosociological studies have been conducted in Arctic tundra, including the Svalbard archipelago, but none have yet attempted to distinguish differences in plant communities associated with colonies of different bird species (e.g., Vanderpuye et al., 2002; Hodkinson et al., 2003; Węgrzyn and Wietrzyk, 2015). Indeed, some studies have concluded that the observed large variation of plant communities near intense sources of ornithogenic nutrients such as bird cliffs remains uninvestigated (Eurola and Hakala, 1977; Elvebakk, 1994).

We have previously addressed the soil chemistry in the vicinity of the two major types of Arctic seabird colonies, i.e., of planktivorous little auks and piscivorous Brunnich’s guillemots and black-legged kittiwakes, based on the same study areas and sample plots set as presented in this paper. These studies demonstrated that the soil phosphate content pH were much higher near the piscivorous colony (Zwolicki et al., 2013). This leads to the hypothesis that the colony-specific soil chemistry could lead to the formation of distinctive plant communities, known as ‘ornithogenic’ or ‘bird cliff vegetation’ (Eurola and Hakala, 1977), in terms of their species composition and functioning.

Therefore the aim of this study was to compare the influence of planktivorous and piscivorous seabird colonies on arctic plant communities by testing the following hypotheses:

(1) Plant communities developing in the vicinity of planktivorous and piscivorous seabird colonies will differ as a result of physical and chemical differences in soil properties around these colonies.
(2) The abundance of particular plant species and the distribution of plant communities will change along the guano deposition gradient at different rates around the two colony types.

## Materials and Methods

### Study Area

**FIGURE 1:**
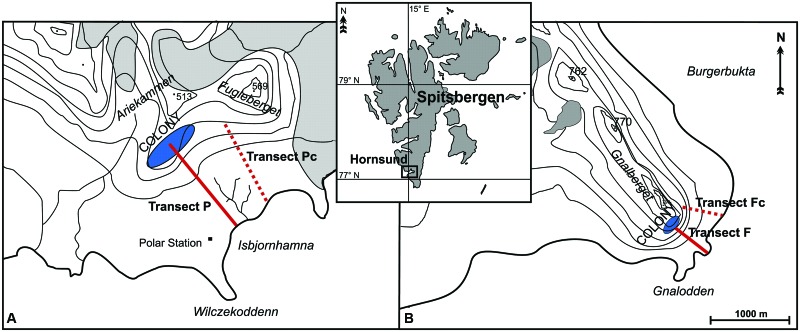
**Study area and the location of transects: the vicinity of the planktivorous little auk colony (A)**, and that of the piscivorous mixed Brunnich’s guillemot and kittiwake colony **(B)**. Map based on Zwolicki et al. (2013).

The research was conducted in the summer months of 2005 and 2006, on the northern coast of Hornsund fjord (south-west Spitsbergen). Two areas influenced by two large seabird colonies were investigated (**Figure 1**):

(1) The vicinity of a planktivorous little auk colony situated on Ariekammen mountain (77°00’N 15°31′E) (**Figure 1A**). The area consisted of a gentle talus slope (inclination 35–45°), that became near-horizontal tundra approaching the seashore (a ca. 1000 m transect).
(2) The area stretching between a mixed colony of piscivorous Brunnich’s guillemots and kittiwakes situated on Gnålberget cliff (77°01′N 15°52′E), and the ca. 500 m distant seashore (**Figure 1B**). The inclination of the scree-covered slope was 40–50° directly under the cliff, flattening to almost horizontal ground at the coast.

Both colonies are long-established and of similar size, each consisting of ca. 10,000 breeding pairs (Isaksen, 1995). Typical simple Arctic soils, mostly gleysols and regosols, occur in the study areas, ranging in depth from 15 to 20 cm (Fischer and Skiba, 1993).

Two colony transects were defined within both study areas, starting from the zone of the highest colony impact (the center of the little auk colony and the foot of the guillemot/kittiwake nesting cliff), running down the slope and ending on the seashore (**Figure 1**). Both transects were exposed to the south-east and covered an altitudinal range from sea level to ca. 200 m asl. The transects consisted of 10 (piscivorous colony) and 12 (planktivorous colony) sample plots (each 160 cm × 160 cm). A greater proportion of plots were situated in the vicinity of each colony, where the greatest variety of vegetation zones was present, than in the more distant and homogeneous coastal area. The little auk colony covered a large area of the relatively gentle slope and had a less clear-cut boundary than was the case for the cliff colony. Sampling plots were located at increasing distance from the starting points (plot 1), as follows: plot 2 (6 m), 3 (15 m), 4 (29 m), 5 (49 m), 6 (79 m), 7 (125 m), 8 (193 m), 9 (296 m), 10 (449 m), 11 (680 m), and plot 12 (1026 m) (the latter two plots only for the little auk colony).

In both study areas, control transects were defined in topographically similar locations but not under the routine flight route of the seabirds, hence experiencing negligible ornithogenic impact (Zwolicki et al., 2013). Eleven (piscivorous colony) and 12 (planktivorous colony) plots were designated along the control transects following the principle described above. The transects situated close to the little auk colony were annotated as P (planktivorous) and Pc (control), while those situated under the fish-eaters’ colony were annotated as F (piscivorous) and Fc (control).

### Guano Deposition Measurements

Along all four transects guano deposition was assessed using black plastic sheets (150 cm × 150 cm) placed next to each sampling plot for 24 h (extended or shortened depending on weather conditions, in the range of 20–36 h, in a few cases). After exposure, a digital photograph of each sheet was taken (Canon PowerShot A95, resolution 5.0 million pixels). Then the sheets were cleaned and re-exposed for the next 24 h cycle. Depending on logistics, wind and precipitation conditions the total lengths of exposition on the four transects were as follows: *P* = 144 h, *P*c = 144 h, *F* = 216 h, *F*c = 24 h. The guano-covered area in each photograph was analyzed using SigmaScan Pro 5.0.0 software.

In order to provide an accurate estimate of guano deposition (dry mass) from the photographs obtained, we performed an initial calibration. We exposed stiff plastic sheets (150 cm × 150 cm) covered with a thin plastic film of known mass. After these sheets were exposed and photographed, the plastic films were removed, dried and re-weighed to obtain the dry mass of guano. Regression equations for guano area to mass relation were calculated for each study area separately [planktivorous colony, *y* = 0.003 *x*, *R*^2^ = 0.7, *N* = 31; piscivorous colony, *y* = 0.008 *x*, *R*^2^ = 0.7, *N* = 10; where: *x* – area covered by guano (cm^2^), and *y* – guano dry mass (g)].

### Physical and Chemical Analyses of Soil

Soil samples were collected from three points on the same diagonal of each sampling plot (one from the center and two from the corners of the plot) (*N* = 123). Samples were taken from the soil surface layer to a depth of 10 cm using a shovel. Each sample contained about 500 cm^3^ of soil. Larger stones were avoided or removed during sampling. Soil samples were prepared for analysis immediately after collection in the field laboratory. Each sample was divided into three subsamples of 80 cm^3^ each, weighed to the nearest 0.1 g and the following were assessed:

(1) Soil dry mass (%) was measured by oven-drying (60°C) a sub-sample until constant mass. The % soil dry mass was calculated from the difference between the initial and final masses.
(2) Soil conductivity (μS cm^-1^) and pH – Soil samples of 80 cm^3^ were mixed with 160 cm^3^ of distilled water. The mixture was shaken for ca. 20 min and then filtered through a sieve (0.5 mm mesh). The conductivity and pH were quantified in the filtrate using a pH/conductivity/salinity meter CPC-401 (Elmetron).
(3) Nitrate (NO_3_^-^), ammonium (NH_4_^+^), potassium (K^+^) and phosphate (PO_4_^3-^) content (mg 1000 g^-1^ soil dry mass) – Soil samples of 80 cm^3^ were mixed with 200 cm^3^ 0.03 N acetic acid, and left for ca. 60 min while being shaken regularly. The solution was then filtered through a sieve (0.5 mm mesh) and filter paper (MN 640 w, Macherey–Nagel Φ = 125 mm). The filtrate was analyzed using a photometer LF205 following standard procedures (Cygański, 1994).

### Stable Isotope Analyses

To assess δ^15^N signatures and total nitrogen content in soil we used sub-samples left after drying and mass assessment (*N* = 117). They were sieved through a 0.25 mm mesh to remove stones and larger plant debris, and ground with a vibrating mill (LMW-S, Testchem) to a grain size of less than 0.03 mm diameter.

In the case of plant tissues, we collected three samples from the above-ground parts of common vascular plants (such as *Poa alpina*, *Deschampsia alpina*, *Cochlearia groenlandica*, *Cerastium arcticum*, *Saxifraga oppositifolia*, *Salix polaris*) (*N* = 350) and mosses (*N* = 91) from each sample plot (not less than 5 mg dry mass in each sample). After collection they were manually cleaned of contaminants such as guano, soil particles, etc., dried at 40–60°C to a constant mass and ground with a vibrating mill. The results from different species were averaged per plot for the vascular plants and mosses.

Prior to the isotopic analyses the soil samples were cleaned of lipids using 4 ml of cyclohexane per 50 mg of soil. After this a small amount of each soil and plant sub-sample (1–2 mg, weighed with a microbalance, precision 0.001 mg) was packed into a tin capsule. Nitrogen isotope ratios were determined by a continuous flow mass spectrometer (Thermo Fisher, Delta V Advantage) coupled to an elemental analyzer (Thermo Fisher, Flash EA 1112) at the University of La Rochelle (France). Results were expressed in the conventional δ^15^N notation, according to the equation: δ X = (*R*_sample_ *R*_standard_^-1^ - 1) 1.000 (‱), where *R*_sample_ was the stable isotope ratio ^15^N/^14^N in the analyzed sample, and *R*_standard_ was the stable isotope ratio ^15^N/^14^N in the reference material, i.e., atmospheric N_2_ (Kelly, 2000).

### Vegetation Abundance and Species Composition

Within each sampling plot we identified vascular plant and moss species, and visually estimated the percentage contributions of the species and the entire groups (vascular plants and mosses) to total vegetation cover. Samples of algae and lichens were collected and identified in the laboratory without abundance measurements.

Nomenclature for vascular plants and moss species follows Elvebakk and Prestrud (1996). Taxonomy of Chlorophyta was based on Hoek et al. (1995), that of Cyanobacteria on Anagnostidis and Komárek (1988) and Komárek and Anagnostidis (1999) [species names updated after Komárek (2016)], and that of lichens on Kristinsson et al. (2010).

The identified vegetation groups (see below) were defined as communities (Putman, 1994), and we used the two dominant species names to create each community’s name.

### Statistical Analysis and Data Management

To distinguish vegetation groups (communities) and to identify cutoff values of the related environmental variables a linkage tree analysis (LINKTREE) with SIMPROOF test was performed (Clarke et al., 2008). Differences in vegetation and soil physico-chemical properties between the five LINKTREE groups (G1–G5) identified were examined with one-way ANOSIM (analysis of similarities, with the Monte Carlo permutation test). These analyses were run on log-transformed data [*x′* = log (*x* + 1)] to reduce the influence of dominant species. Soil variables were additionally standardized because parameters represented different units and scales. Similarity percentages analysis (SIMPER) was used to define the contribution of each taxon to dissimilarities between the distinguished groups and to described average percentage cover (AC, %) and average similarity (AS) within each group. LINKTREE, ANOSIM, and SIMPER were run in Primer 6.1.5 (Clarke and Gorley, 2006).

To explore theoretical environmental gradients in the data, and to calculate the variability explained by specific environmental variables, unconstrained Detrended Correspondence Analysis (DCA) was used. Canonical Correspondence Analysis (CCA) was used to examine the influence of soil parameters and guano deposition on vegetation variability. The influence of guano deposition on plants was tested separately for each colony, because our previous study showed that the same amount of guano from the different bird species influenced soil chemistry differently (Zwolicki et al., 2013). To identify which of the factors significantly influenced the CCA model, a Monte Carlo test (with 499 permutations) was performed. For multiple comparisons we used Holm’s correction to control the family-wise type I errors (Holm, 1979). The efficiency of the environmental variable(s) in explaining the non-random variability existing in the data (%) was calculated by dividing the percentage variability explained by a given environmental factor by that explained by the first four axes of DCA (ter Braak and Smilauer, 2012). All ordination techniques used log-transformation [*x′* = log (*x* + 1)] in order to normalize the data.

To explore individual responses of selected plant species to guano deposition level we employed General Linear Models (GLM) with Akaike Information Criterion (AIC) to find the best fit of the model. DCA, CCA, and GLMs were run in Canoco 5 (ter Braak and Smilauer, 2012).

We compared the identified vegetation groups with each other using parametric ANOVA with Welch’s correction (because of unequal variances between groups) and *post hoc* Tukey’s test, using the STATISTICA 9.0 package (StatSoft Inc, 2010).

## Results

### Vegetation Communities

**FIGURE 2:**
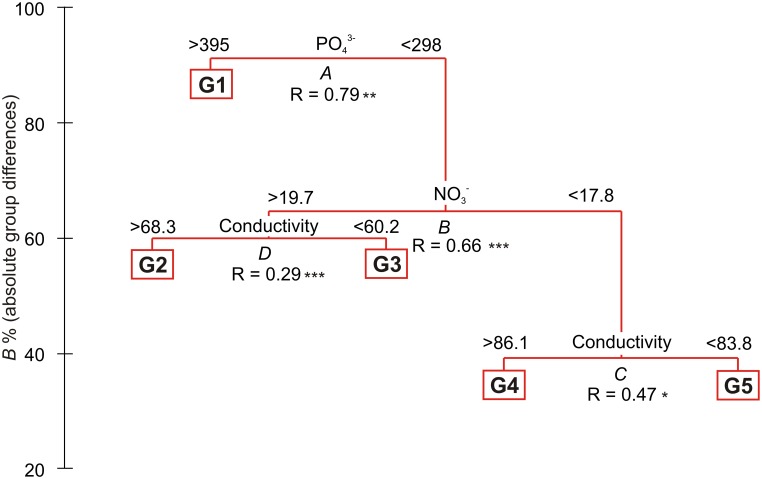
**Diagram of linkage tree analysis (LINKTREE) showing distinct clustering of plots based on vegetation composition constrained by inequalities on environmental variables**. For each split ANOSIM *R*-value was calculated with significance levels ^∗∗∗^*p* < 0.001, ^∗∗^*p* < 0.01, ^∗^*p* < 0.05. All distinguished groups were confirmed by SIMPPOF test, *p* < 0.001. For detailed description of groups see **Table 1**; Supplementary Table S1.

Within all four transects 75 taxa of plants were determined, including 27 vascular plants and 48 mosses (**Table 1**; Supplementary Table S1). Four successive two-way divisions split all 45 sample plots to the final five groups each significantly differing composition (LINKTREE and SIMPROF test, *p* < 0.05; **Figure 2**; **Tables 1** and **2**; Supplementary Figure S1). The primary significant division (A) with the highest absolute difference (*B*% = 91) distinguished a group of samples containing the first seven plots below the piscivorous colony (group G1; **Figure 2**). This division was linked to a high concentration of phosphates (>395 mg 1000 g^-1^ soil dry mass). The next division (B, *B*% = 65) was connected to the concentration of nitrates and separated the G2 and G3 groups (NO_3_^-^ > 19.7 mg 1000 g^-1^), containing plots from the planktivorous colony transect and one plot (8) from the piscivorous colony, from groups G4 and G5 (NO_3_^-^ < 17.8 mg 1000 g^-1^) (**Figure 2**; **Table 2**). The latter two groups included plots from the control transects and the most distant plots from both colony transects. The last two divisions (C, *B*% = 39, and D, *B*% = 60) were based on conductivity levels and separated groups G2 (>68.3 μS cm^-1^) from G3 (<60.2 μS cm^-1^), and G4 (>86.1 μS cm^-1^) from G5 (<83.8 μS cm^-1^) (**Figure 2**).

**Table 1:** Characteristics of the five distinguished LINKTREE groups (cf. **Figure 2**) with average percentage cover (AC, %) and average similarity (AS) within each group, based on SIMPER analysis.

	LINKTREE groups									
	Group G1		Group G2		Group G3		Group G4		Group G5	
	
Community	*C. groenlandica– P. alpina*		*D. alpina– C. arcticum*		*S. uncinata–S. stramineum*		*S. uncinata– S. oppositifolia*		*S. uncinata– S. polaris*	
N plots	7		6		4		7		18	
N vascular plants	9		13		9		12		22	
N mosses	11		17		18		24		31	
N algae	17		18		13		10		30	
N lichens	0		3		9		14		40	
N total taxa	44		57		53		67		141	
Average similarity	38.96		25.45		15.76		22.17		44.76	

**Taxa**	**AC**	**AS**	**AC**	**AS**	**AC**	**AS**	**AC**	**AS**	**AC**	**AS**

*Sanionia uncinata*	0.01	0	17.83	4.35	37.04	8.9	26.59	11.43	38.6	34.65
*Cerastium arcticum*	12.14	2.15	24.33	8.57	20.6	3.49	0.91	0.28	0.09	0.03
*Cochlearia groenlandica*	42.14	28.62	1.03	0.49	0.6	0.08	0.06	0.07	0.03	0.01
*Saxifraga oppositifolia*	0	0	0	0	4.8	0	21.43	7.05	7.76	5.15
*Deschampsia alpina*	0	0	33.33	7.68	0	0	0.19	0.07	0.07	0.01
*Poa alpina*	16.01	6.55	6.72	1.82	6.4	0.85	0	0	0	0
*Salix polaris*	0	0	7.5	0.36	4	0	0	0	10.25	4.11
*Straminergon stramineum*	0	0	0	0	20.8	1.28	0	0	0.4	0

*Taxa listed (eight most abundant species) according to the average total coverage. In each group, AC and AS values of two dominant species are marked with boxes. Complete data on all 75 taxa are presented in Supplementary Table S1)*.

**Table 2:** Arrangement of sample plots and LINKTREE group (cf. Figure 2; Table 1) membership within four studied transects.

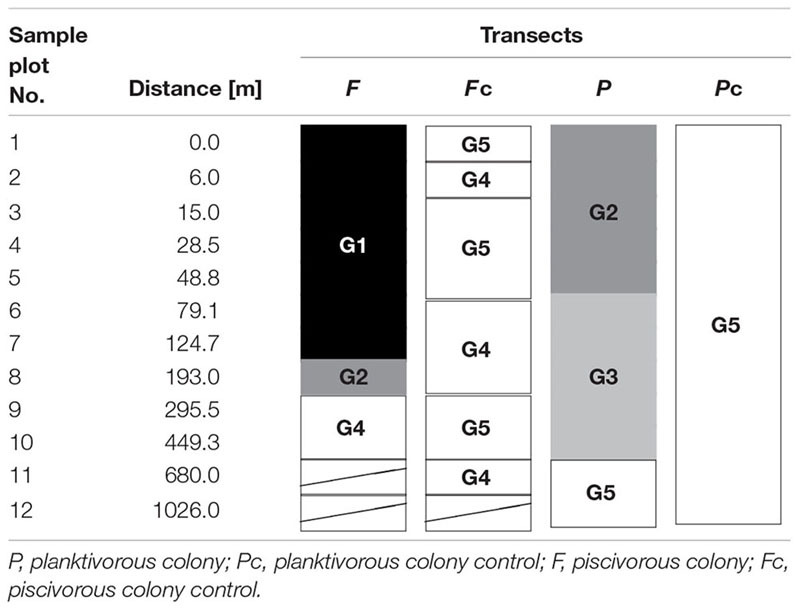

*P, planktivorous colony; Pc, planktivorous colony control; F, piscivorous colony; Fc, piscivorous colony control.*

**FIGURE 3:**
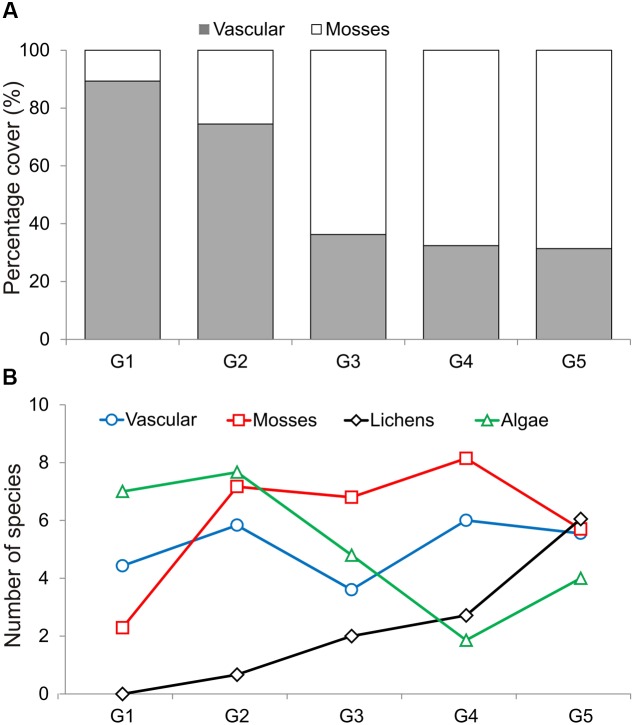
**Mean percentage cover of vascular plants and mosses (cumulated to 100%) (A)**, and mean number of species of vascular plants, mosses, lichens and algae **(B)** found in the five LINKTREE groups.

Differences between communities were observed in the mean cover of vascular plants and mosses, and also in the mean number of algae and lichen species (ANOVA Welch test; *p* < 0.01, *post hoc*, *p* < 0.01, **Figure 3**). The highest vascular plant cover and the lowest moss cover were found in G1 and G2, while the opposite was found in G3, G4, and G5. The largest mean number of algae species was found in G1 and G2, and the lowest in G4. No lichens occurred in G1, and the number of lichen species increased with subsequent groups, reaching the highest value in G5 (**Table 1**; Supplementary Table S2). The lowest numbers of species of vascular plants, mosses, and lichens were observed in G1 (*N* = 44 in total), and the highest (also the number of algae) in G5 (*N* = 141). G5 was the most homogeneous with the highest average internal similarity (AS = 44.8), while the lowest similarity (15.8) was found in G3 (**Table 1**).

Associated with the piscivorous colony, the G1 community (*Cochlearia groenlandica–Poa alpina*) had the highest average cover and similarity of *C. groenlandica* (AC = 42.1%, and AS = 28.6) and *P. alpina* (16.0% and 6.5, respectively), and relatively high abundance of *Saxifraga caespitosa* and *C. arcticum* (**Table 1**). These vascular plant species were accompanied by the green alga *Prasiola crispa* and the cyanobacteria *Phormidium autumnale*, *Borodinellopsis texensis. Haematococcus pluvialis, Excentrosphaera viridis*, *Ulothrix variabilis*, and *Woronichinia compacta* (Supplementary Table S3).

The G2 and G3 groups associated primarily with the planktivorous colony were characterized by relatively high cover of *C. arcticum* (24.3 and 20.6%, respectively) (**Table 1**). The G2 community (*Deschampsia alpina–Cerastium arcticum*) was dominated by *D. alpina* (AC = 33.3), and *Saxifraga hyperborea* was relatively abundant there (4.8). Only in this assembly was *Philonotis tomentella* recorded. In terms of algae, G2 was distinguished by the presence of *Chlamydomonas nivalis*, *Chroococcus turgidus, Leptolyngbya* cf. *foveolarum*, the genus *Trochiscia*, and *P. autumnale* (Supplementary Table S3). Amongst the lichens two *Physcia* species and *Xanthoria candelaria* were found exclusively in this group (Supplementary Table S2). G3, G4, and G5 included communities dominated by bryophytes, especially *S. uncinata* (AC = 37.0, 26.6, and 37.6, respectively, **Table 1**).

The *Sanionia uncinata–Straminergon stramineum* community (G3) was distinctive due to the high cover of both dominant species (AC = 37.04 and 20.8, respectively), and relatively high cover of *Plagiomnium ellipticum* (AC = 4.0). Characteristic algae species recorded in this community, besides those identified in G1 and G2, were *Oscillatoria tenuis*, *Phormidium favosum* and *Scotiellopsis terrestris* (Supplementary Table S3). Most of the lichen species belonged to the genus *Cladonia* (Supplementary Table S2).

The G4 community (*Sanionia uncinata–Saxifraga oppositifolia*) was characterized by the highest abundance amongst the groups of *S. oppositifolia*, as well as the presence of *Ceratodon purpureus*. The G5 group (*Sanionia uncinata–Salix polaris*) was distinctive due to the highest abundance of *S. polaris* in comparison with the other communities. In G4 and G5 the highest numbers of cyanobacteria and algae species were noted. Numerous species of the genera *Gloeocapsa*, *Calothrix*, *Nostoc*, *Tolypothrix*, *Scytonema*, *Dichothrix*, and also heterocystous filamentous members of Nostocales (including *Calothrix, Nostoc, Tolypothrix*, and *Scytonema*) were present. G4 and, particularly, G5 included the largest number of lichen species, mostly from the genera *Bacidia, Caloplaca*, and *Lecanora* (Supplementary Table S3).

### Soil Characteristics of Vegetation Communities

**FIGURE 4:**
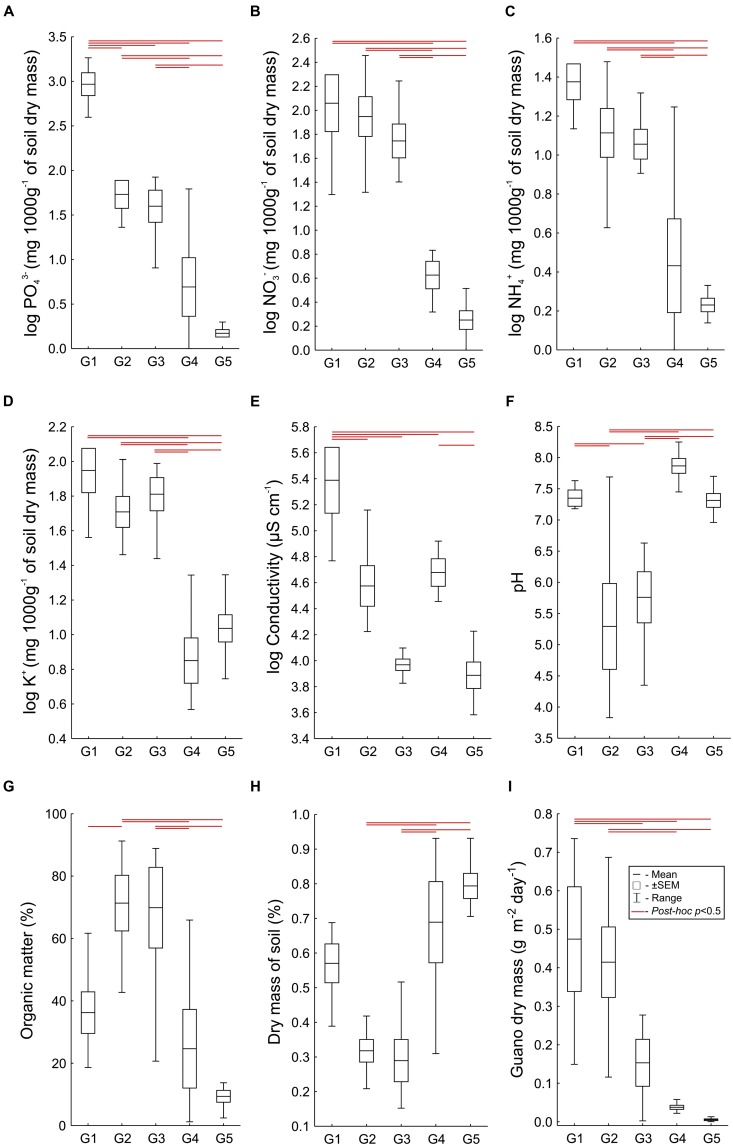
**Levels of physical and chemical soil parameters (A-H)** and guano deposition level **(I)** in each of the LINKTREE vegetation groups. Significant differences between pairs of groups are indicated by horizontal lines (*p* < 0.01).

Analysis of similarity between the LINKTREE groups, based on all the physical and chemical parameters measured, revealed significant differences between each group (ANOSIM; *R* = 0.71, *p* = 0.001, all *post hoc* tests, *p* < 0.01, with the exception of G2 vs. G3, *p* = 0.48). Detailed comparisons executed separately for each physical and chemical soil property also revealed significant differences between the groups (all Welch’s ANOVA tests, *p* < 0.01; in all the *post hoc* pairwise comparisons where the differences were found *p* < 0.01), and clear environmental gradients in subsequent vegetation units for most of the parameters (**Figure 4**). In general, the highest values of all the measured ion concentrations were found in G1 and G2, and they decreased in G3 and G4, reaching the lowest values in plots from G5 (**Figures 4A–D**). Phosphate and nitrate concentrations, and conductivity, the parameters identified by LINKTREE analysis as important separating factors between groups, clearly and significantly differed between these groups (**Figures 4A,B,E**). Significant differences between G1 and G2 were found also in ammonium concentration, pH, conductivity and soil dry mass, with higher values observed in G1 (**Figure 4C**). The lowest values of soil dry mass were found in G2 and G3, which corresponded with the highest content of organic matter in these groups (**Figures 4G,H**). Guano deposition was highest in G1 and G2, and the lowest in G5, although there were no significant differences between G1 and G2, and between G4 and G5 (**Figure 4I**).

### Stable Nitrogen Isotope and Total Nitrogen Content

**FIGURE 5:**
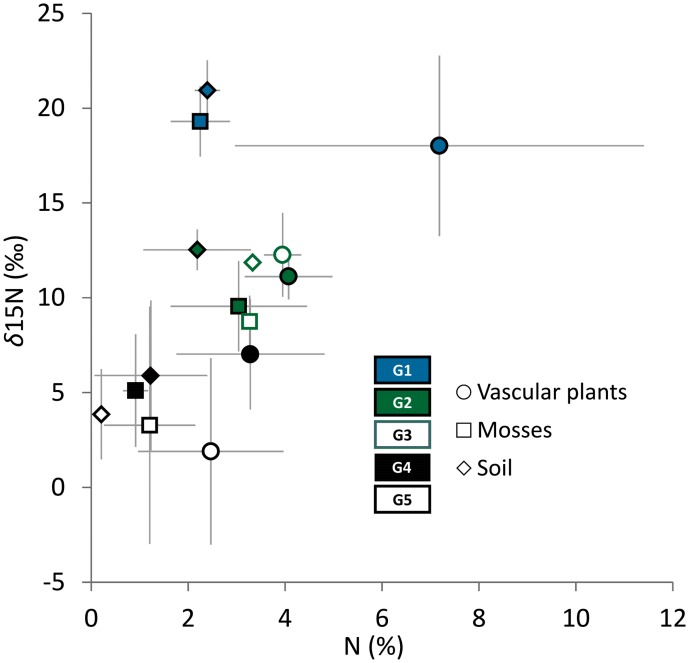
**Relationships between δ^15^N (‱) and total nitrogen content (%) in vascular plants, mosses and soil samples within the LINKTREE groups G1–G5**. Points represent mean values and error bars show standard deviations. For *post hoc* pairwise comparisons see Supplementary Table S4.

The five LINKTREE groups showed clear separation in terms of total nitrogen contents and stable isotope ratios in soil and vascular plant and moss tissues (Welch’s ANOVA *p* < 0.01) (**Figure 5**). The highest total N and δ^15^N values both in soil and plant tissues were found in G1, with intermediate values characterizing G2 and G3 (no significant differences between these two groups,), and the lowest levels recorded in G4 and G5 (see detailed results of *post hoc* comparisons in Supplementary Table S4). Differences in total nitrogen content in almost all comparisons followed the differences in stable isotope signatures (**Figure 5**).

### Environmental Gradient Identification

**FIGURE 6:**
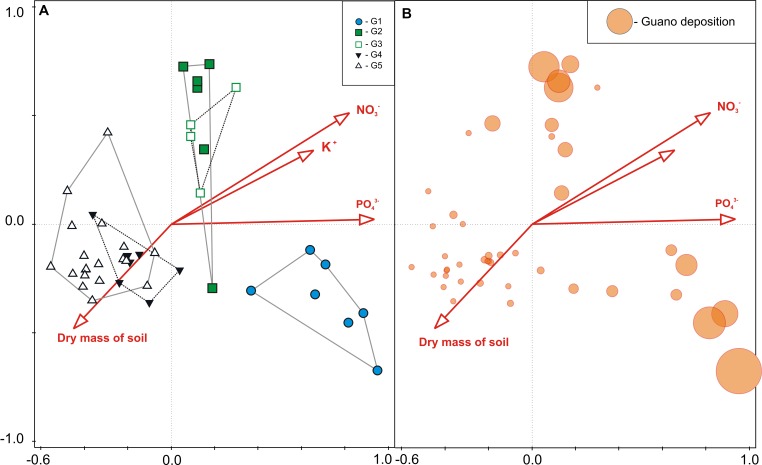
**(A)** CCA sample plots ordination based on vegetation (vascular plants and mosses) community composition, indicating the LINKTREE groups G1–G5 and relationship with significant soil parameters (see **Table 3**), and **(B)** guano deposition level indicated by circle size, within each sample plot.

Considering all 45 plots together, all seven soil parameters tested explained all of the variability (100% efficiency) in vegetation composition, and four of them were statistically significant (CCA, Monte Carlo permutation test, *p* = 0.014). The most important variable was PO_4_^3-^ concentration, which explained 38.9% of variation, while those of secondary importance were soil dry mass, NO_3_^-^ and K^+^ concentrations (**Table 3**). The constrained ordination diagram shows clear separation of the groups in terms of vegetation composition in relation to the soil parameters (**Figure 6A**), despite a similar guano deposition level (**Figure 6B**).

**Table 3:** Total and explainable conditional effects of different environmental variables on plant community composition (CCA) (cf. **Figure 4**).

Response data	Explanatory variable	Total variation %	Efficiency^1^	*pseudo-F*	*p(adj)*^2^
All transect	PO_4_^3-^ log	11.2	38.9	5.3	0.014
	Dry mass of soil	6.2	21.5	3.1	0.014
	NO_3_^-^ log	5.4	18.7	2.8	0.014
	K^+^ log	4.0	13.9	2.1	0.014
	pH	2.9	10.1	1.6	0.090
	NH_4_^+^ log	1.8	6.2	1.0	0.900
	Conductivity	1.4	4.9	0.7	0.900
Plankton-eaters (*P* and *P*c combined)	Guano deposition	13.1	57.4	4.5	0.002
Fish-eaters (*F* and *F*c combined)	Guano deposition	11.3	54.0	3.4	0.002

*^1^The result of comparison with the unconstrained model, ^2^Adjusted by the Holm correction*.

**FIGURE 7:**
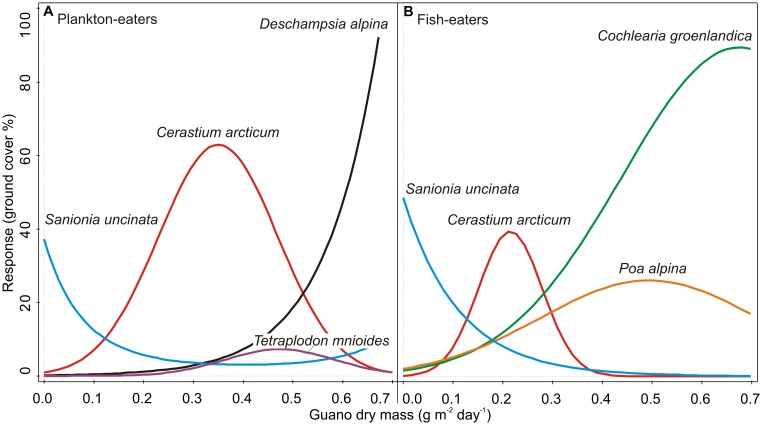
**GLM species response curves to guano deposition level: (A)** planktivores (*P* and *P*c combined), **(B)** piscivores (*F* and *F*c combined). Detailed results of the models are given in **Table 4**.

Guano deposition was tested independently for each colony (see Statistical Analysis and Data Management) and had statistically significant effects on the plant community composition in both cases (CCA, Monte Carlo permutation test *p* < 0.01). It explained 57.4% of the variation of plants close to the planktivorous colony (*P* and *P*c combined), and 54.0% near the piscivorous colony (*F* and *F*c combined) (**Table 3**). GLM analysis revealed that, with increasing planktivore guano deposition the percentage cover of *D. alpina* increased (**Table 4**; **Figure 7A**), while a high level of piscivore guano deposition promoted *C. groenlandica* and *P. alpina* (**Figure 7B**). In both areas *C. arcticum* demonstrated a unimodal response along the guano deposition gradient, and *S. uncinata* showed a negative relationship with guano supply (**Figure 7**).

**Table 4:** Response of specific plant species to guano deposition level (GLM model; for response curves, see **Figure 7**).

Study area (colony)	Species	Type	*R*^2^ (%)	*F*	*p*
Plankton-eaters (*P* and *P*c combined)	*Cerastium arcticum*	Quadratic	81.6	35.6	<0.001
	*Deschampsia alpina*	Linear	89.5	149.4	<0.001
	*Sanionia uncinata*	Quadratic	34.1	5.9	0.009
	*Tetraplodon mnioides*	Quadratic	94.3	171.3	<0.001
Fish-eaters (*F* and *F*c combined)	*Cerastium arcticum*	Quadratic	81.3	28.9	<0.001
	*Cochlearia groenlandica*	Quadratic	78.0	22.1	<0.002
	*Poa alpina*	Quadratic	33.8	4.3	0.031
	*Sanionia uncinata*	Linear	37.0	9.8	0.006

## Discussion

### Arctic Vegetation Response to Seabird Influence

Our data strongly suggest that the colony of planktivorous little auks and the mixed colony of piscivorous guillemots and kittiwakes affect the neighboring vegetation in different ways. This was manifested by the development of specific plant communities, where the ornithogenic vegetation could be subdivided into different subtypes based on the diet of the respective influencing bird colonies.

The distinction between the two bird cliff vegetation sub-types was most evident between the G1 and G2 groups. G1 (*Cochlearia groenlandica–Poa alpina*) communities were located in the immediate vicinity of the piscivorous colony (from the bird cliff to 125 m down the slope), while the G2 community (*Deschampsia alpina–Cerastium arcticum*) occurred within the little auk colony (down the slope to ca. 50 m from the colony center). The G1 community was characterized by the domination of one species, *C. groenlandica*, resulting in low diversity and the lowest numbers of vascular plant, moss and lichen species compared to all the other communities. Domination of *C. groenlandica* in ornithogenic plant communities seems to be characteristic of piscivorous colonies in many places in the Svalbard archipelago, while in case of planktivorous little auk colonies this species is also present but never as a dominant (Eurola and Hakala, 1977; Odasz, 1994; Rønning, 1996; Zwolicki et al., 2016). Within G2 community, the co-domination of two vascular plant species, *D. alpina* and *C. arcticum*, as well as one bryophyte species, *S. uncinata*, was observed. Differences between communities were also noticeable in the proportion of species of secondary importance in a community structure. Relatively high abundance of saxifrages, e.g., *S. caespitosa* and *S. cernua*, was distinctive for G1, while in G2 *S. hyperborea*, and the mosses *T. mnioides* and *Cyrtomnium hymenophylloides* were characteristic.

Differences between the colonies were also apparent in species responses to the guano deposition gradient. We found a strong positive correlation between amount of guano and abundance of *D. alpina* (planktivorous colony) and *C. groenlandica* (piscivorous colony). Even *C. arcticum*, which occurred in the vicinity of both colonies and showed full gaussian curves, responded differently to the same amount of guano input (with peak of response curve in 0.35 (guano dry mass g m^-2^ day^-1^) for plankton-eaters when 0.21 for fish-eaters). This suggests a higher tolerance of this plant to little auk than guillemot/kittiwake excreta and qualitative differences between colonies influence.

The LINKTREE analysis indicates that the clear differences in vegetation apparent around the two colonies were probably related to significant differences in soil chemical and physical parameters, which may themselves be related to differences in the birds’ diet and excreta (Gilham, 1956). These differences were further supported by the nitrogen stable isotope signatures, which were significantly higher in samples collected near the piscivore colony. Higher values of δ^15^N in soil were associated with higher levels in vascular plant and moss tissues, indicating that vegetation developing close to the studied colonies uptakes the different nutrients delivered by the seabirds. Because higher phosphate content is present in fish tissues than those of zooplankton (Andersson et al., 1988), the G1 community associated with the piscivore colony was characterized by nearly twice the amount of phosphorus being present in the soil. Large differences were also observed in soil pH, with G2 characterized by being acidic although, at the same time, showing the greatest variation in this parameter. Higher pH beneath the piscivore colony could be very important for the development of the *Cochlearia groenlandica–Poa alpina* community, as it can change the availability of phosphorus and nitrogen, and reduce the negative effects of over-fertilization (Arnesen et al., 2007). Also, differences between communities, related to colony types, were associated with differences in the soil organic matter content, which was higher in the G2 and G3 communities associated with the planktivore’s colony. Thus, the two types of bird colonies favor plants with different environmental requirements, and this was especially evident closest to the colonies. The hypothesis that different colony types encourage the development of different habitats was also strongly supported by the similar values of soil properties found between control transects, as noted previously (Zwolicki et al., 2013). It is also worth noting that fresh birds’ excreta could be poisonous for plants due to high acidity of uric acid, especially in high concentrations. Large penguin colonies that are completely denuded of plants could be an extreme example of such influence (Zwolicki et al., 2015). Also, a direct deposition of feces on leaves could create osmotic stress or even mechanically block the stomata. Future experimental studies concerning controlled environmental conditions, pot, transplant, or common garden experiments could unravel the mechanism of relationship between the type of colonies and plant communities formation.

Eurola and Hakala (1977) studied bird cliff vegetation in Svalbard in the vicinity of colonies occupied by different seabird species, mostly guillemots, kittiwakes and little auks. They described ornithocoprophilous vegetation as *Chrysosplenium tetrandrum–Oxyria digyna* type and divided it into three subtypes: meadow, ledge, and boulder communities. However, their study did not attempt to match plant community differentiation with bird species or diet. This distinction has been overlooked till now possibly because plant communities’ composition changes with other important environmental variables connected to, e.g., geographical localization. Such comparison of five locations across the Svalbard archipelago revealed that plant communities occurring near little auk colonies were locally mostly dependent on birds fertilization intensity, but, more than that, their geographical distribution was an important factor modifying plants’ community structure (Zwolicki et al., 2016). Therefore, the comparison between types of colonies could be verified only in similar locations, as we present in our research, where the same pool of species could be recruited to local communities.

Despite the different effects the two colony types had on their surrounding vegetation, there were also some common characteristics of their influence. Guano deposition had similarly high importance for plant community composition variability, which could indicate similar impact strength on vegetation development beneath both colonies. Guano deposition was similarly the most important factor influencing physical and chemical parameters of the soil, where it had similar importance for both colonies (Zwolicki et al., 2013).

Both ornithogenic community sub-types were characterized by high abundance of grasses (*D. alpina*, *P. alpina*, and *F. rubra*), the plant group previously proposed to be the primary creators of meadow communities in the Arctic (Elvebakk, 1994). Nitrophilous dicotyledonous species, such as *C. arcticum* and *C. groenlandica*, commonly occurring within G1 and G2, are also frequently recorded in the vicinity of seabird colonies in the Arctic (e.g., Odasz, 1994; Aiken et al., 1999; Brysting et al., 2001). Areas closest to both colonies hosted an unique composition of green algal species, including the ornithocoprophilous *P. crispa* and *P. autumnale*, and the highly eutrophic cyanobacterium *Pseudanabaena* sp. (Matuła et al., 2007; Richter et al., 2009). However, the detailed qualitative and quantitative studies conducted in the same locations revealed that cyanobacterial and algal assemblages were also differentiated by the colony type (Pietryka et al., 2016). The ornithogenic communities G1 and G2 were largely devoid of lichens, except for the ornithocoprophilous *Physcia* spp. and *X. candelaria* near the little auks colony (Øvstedal, 2009; Wirth, 2010).

A decline in vascular plant abundance and increase in mosses, as well as an increase in the total number of vascular plant, moss and lichen species, were observed with progression through the G1–G5 communities, with clear zonation with distance from the colonies. The immediate vicinity of both studied colonies favored fast growing and short-lived species (e.g., *C. arcticum* lives around 10 years, *C. groenlandica* about 5 years), while those growing further from the colony were often much longer-lived (e.g., *Salix* sp. about 59 years) (Callaghan and Emanuelson, 1985). This zonation pattern has been recorded in previous studies (cf. Croll et al., 2005; Ellis, 2005; Zwolicki et al., 2015). Plant communities developing in polar areas without or with a relatively low ornithogenic impact experience a chronic shortage of nutrients and are dominated by mosses, lichens, and stress-resistant vascular plants, with long-lived leaves and high nutrient use efficiency (Grime, 1979; Chapin, 1980). In our study, this strategy was represented primarily by *S. oppositifolia* and *S. polaris*. These species have been described as early colonizers (*S. oppositifolia*) and as plants typical of the late succession stage (*S. polaris*), and their abundances decreased with nutrient inflow (Hodkinson et al., 2003; Nakatsubo et al., 2010). Nutrient availability determines plant life history strategies, and the ability to respond rapidly to increased nutrient supplies should give a competitive advantage over species adapted to low nutrient levels (Hill et al., 2011). Furthermore, in harsh environmental conditions, stress resistance had greater importance for plants than the ability to compete with other species (Theodose and Bowman, 1997). *Sanionia uncinata–Saxifraga oppositifolia* (G4) and *Sanionia uncinata–Salix polaris* (G5) communities dominated the control transects, representing plants inhabiting poor habitats with no or negligible ornithogenic impact, such as are common on the coast of south-western Spitsbergen (Elvebakk, 1994; Hodkinson et al., 2003; Węrzyn and Wietrzyk, 2015). Perennial dwarf woody shrubs comprising these communities are well adapted to unpredictable resource availability that limits their growth, and are able to store their restricted resources; hence, they are widespread in the Arctic deserts (Grime, 1979; Chapin et al., 1990). Cyanobacterial and algal assemblages from these nutrient-poor habitats were characterized by numerous species, particularly heterocystous cyanobacterial taxa forming the biological soil crust, in which the deficiency of nitrogen and carbon are compensated through biological N_2_-fixation by free living cyanobacteria (Zielke et al., 2005; Matuła et al., 2007; Stewart et al., 2011; Skrzypek et al., 2015). These habitats are also characterized by a large number of lichen species that prefer oligotrophic and dry habitats, such as moribund mosses (*Caloplaca* spp., *Mycobilimbia* spp., *Bilimbia* spp., *Ochrolechia frigida*, *Bacidia bagiettoana*), or rocks (e.g., *Lecanora* spp.; Øvstedal, 2009; Wirth, 2010).

### Implications for the Ecosystem

Ornithogenic fertilization enhances the nutrient levels of soil and available to plants, as assessed by total nitrogen content, reaching its highest levels in communities growing closest to bird colonies (G1, G2, and G3). High nitrogen content in soil favors plant productivity, increases N content in plant tissues, and promotes species of better palatability, such as grasses, constituting an attractive food source for vertebrate herbivores (Van der Wal and Loonen, 1998). This underlies the much more numerous local populations of geese, ptarmigan and reindeer associated with ornithogenic tundra on Svalbard (Van der Wal et al., 2004; Stempniewicz et al., 2007). Mean values of total nitrogen content in vascular plants near the two colonies were similar. However, the piscivore colony, situated on a coastal cliff, fertilizes a smaller area of tundra, whereas the planktivore colony is located further inland and supports a larger area, and therefore a higher number of herbivores (Stempniewicz et al., 2006, 2007; Jakubas et al., 2008).

Recent oceanographic and climatic changes in the Arctic are leading to changes in the structure of marine zooplankton communities. Large copepods (e.g., *Calanus glacialis*), dominant in cold Arctic waters, and favored by planktivorous seabirds such as little auks, are being replaced by smaller counterparts (e.g., *C. finmarchicus*), characteristic of warmer Atlantic waters. This potentially redirects energy flow through the food chain to planktivorous fish and, finally, to piscivorous guillemots and kittiwakes (Stempniewicz et al., 2007; Carstensen et al., 2012; Weydmann et al., 2014). If this results in a change in balance (numbers) between planktivorous and piscivorous seabird populations, one outcome may be to modify the proportion of the ornithogenic vegetation supported by each. Such changes could therefore have important consequences for the structure and functioning of the terrestrial part of the Arctic ecosystem, as exemplified by ornithogenic plant communities reduced to the piscivorous sub-type (Stempniewicz et al., 2007).

## Author Contributions

Conceived and designed the experiments: AZ, LS, and KZ-S. Performed the experiments: AZ, KZ-S, JM, and BW. Analyzed the data: AZ. Contributed reagents/materials/analysis tools: AZ, KZ-S, LS, JM, and BW. Wrote the paper: AZ KZ-S, and LS.

## Conflict of Interest Statement

The authors declare that the research was conducted in the absence of any commercial or financial relationships that could be construed as a potential conflict of interest.The reviewer JT and handling Editor declared their shared affiliation, and the handling Editor states that the process nevertheless met the standards of a fair and objective review.

## References

[B1] Aiken S. G. Dallwitz M. J. Consaul L. L. McJannet C. L. Gillespie L. J. Boles R. L. (1999). *Flora of the Canadian Arctic Archipelago: Descriptions, Illustrations, Identification, and Information Retrieval. Cochlearia officinalis L. subsp. groenlandica (L.) A.E. Porsild. Version: 29th April 2003.* Available at: https://nature.ca/aaflora/data/www/bacoof.htm

[B2] Anagnostidis K. Komárek J. (1988). Modern approach to the classification system of cyanophytes: 3 Oscillatoriales. *Archiv Hydrobiol.* 50 327–472.

[B3] Anderson W. B. Polis G. A. (1999). Nutrient fluxes from water to land: seabirds affect plant nutrient status on Gulf of California islands. *Oecologia* 118 324–332. 10.1007/s004420050733 28307276

[B4] Andersson G. Granelli W. Stenson J. (1988). The influence of animals on phosphorus cycling in lake ecosystems. *Hydrobiologia* 170 267–284. 10.1007/BF00024909

[B5] Arnesen G. Beck P. S. Engelskjøn T. (2007). Soil acidity, content of carbonates, and available phosphorus are the soil factors best correlated with alpine vegetation: evidence from Troms, North Norway. *Arct. Antarct. Alp. Res.* 39 189–199. 10.1657/1523-0430(2007)39[189:SACOCA]2.0.CO;2

[B6] Bédard J. Therriault J. C. Berube J. (1980). Assessment of the importance of nutrient recycling by seabirds in the St. Lawrence Estuary. *Can. J. Fish. Aquat. Sci.* 37 583–588. 10.1139/f80-074

[B7] Bokhorst S. Huiskes A. Convey P. Aerts R. (2007). External nutrient inputs into terrestrial ecosystems of the Falkland Islands and the Maritime Antarctic region. *Polar Biol.* 30 1315–1321. 10.1007/s00300-007-0292-0

[B8] Brysting A. K. Scott P. J. Aiken S. G. (2001). *Caryophyllaceae of the Canadian Arctic Archipelago: Descriptions, Illustrations, Identification, and Information Retrieval. Cerastium arcticum Lange. Version: 29th April 2003.* Available at: http://nature.ca/aaflora/data/www/cacear.htm

[B9] Byzova J. B. Uvarov A. V. Petrova A. D. (1995). Seasonal changes in communities of soil invertebrates in tundra ecosystem of Hornsund, Spitsbergen. *Pol. Polar Res.* 16 245–266.

[B10] Callaghan T. V. Emanuelson U. (1985). “Population structure and processes of tundra plants and vegetation,” in *Population Structure of Vegetation*, ed. While J. (Dordecht: Dr. W. Junk Publishers), 399–439.

[B11] Carstensen J. Weydmann A. Olszewska A. Kwasniewski S. (2012). Effects of environmental conditions on the biomass of *Calanus* spp. in the Nordic Seas. *J. Plankton Res.* 34 951–966. 10.1093/plankt/fbs059

[B12] Chapin F. S. Schulze E. D. Mooney H. A. (1990). The ecology and economics of storage in plants. *Annu. Rev. Ecol. Syst.* 21 423–447.

[B13] Chapin F. S. III (1980). The mineral nutrition of wild plants. *Annu. Rev. Ecol. Syst.* 11 233–260.

[B14] Clarke K. R. Gorley R. N. (2006). *User Manual/Tutorial.* Plymouth: Primer-E Ltd, 93.

[B15] Clarke K. R. Somerfield P. J. Gorley R. N. (2008). Testing of null hypotheses in exploratory community analyses: similarity profiles and biota-environment linkage. *J. Exp. Mar. Biol. Ecol.* 366 56–69. 10.1016/j.jembe.2008.07.009

[B16] Croll D. A. Maron J. L. Estes J. A. Danner E. M. Byrd G. V. (2005). Introduced predators transform subarctic islands from grassland to tundra. *Science* 307 1959–1961. 10.1126/science.1108485 15790855

[B17] Cygański A. (1994). *Chemiczne Metody Analizy Ilościowej.* Warszawa: Wydawnictwo Naukowo-Techniczne.

[B18] del Hoyo J. Elliott A. Sargatal J. (1996). *Handbook of the Birds of the World. Vol. 3: Hoatzin to Auks*. Barcelona: Lynx editions.

[B19] Ellis J. C. (2005). Marine birds on land: a review of plant biomass, species richness, and community composition in seabird colonies. *Plant Ecol.* 181 227–241. 10.1007/s11258-005-7147-y

[B20] Ellis J. C. Bellingham P. J. Cameron E. K. Croll D. A. Kolb G. S. Kueffer C. (eds) (2011). “Effects of seabirds on plant communities,” in *Seabird Islands: Ecology, Invasion, and Restoration*, eds Mulder C. P. A., (New York, NY: Oxford University Press), 135–176.

[B21] Ellis J. C. Farińa J. M. Witman J. D. (2006). Nutrient transfer from sea to land: the case of gulls and cormorants in the Gulf of Maine. *J. Anim. Ecol.* 75 565–574. 10.1111/j.1365-2656.2006.01077.x 16638009

[B22] Elvebakk A. (1994). A survey of plant associations and alliances from Svalbard. *J. Veg. Sci.* 5 791–802. 10.2307/3236194

[B23] Elvebakk A. Prestrud P. (1996). *A Catalogue of Svalbard Plants, Fungi, Algae, and Cyanobacteria. Skrifter-Norsk Polarinstitutt 198*, (Tromsø: Norsk polarinstitutt), 395.

[B24] Erskine P. D. Bergstrom D. M. Schmidt S. Stewart G. R. Tweedie C. E. Shaw J. D. (1998). Subantarctic Macquarie Island–a model ecosystem for studying animal-derived nitrogen sources using 15N natural abundance. *Oecologia* 117 187–193. 10.1007/s004420050647 28308485

[B25] Eurola S. Hakala A. V. K. (1977). The bird cliff vegetation of Svalbard. *Aquilo Ser. Bot.* 15 1–18.

[B26] Fischer Z. Skiba S. (1993). Some remarks about bioenergetic aspects of tundra soil. *Pol. Polar Res.* 14 345–354.

[B27] García L. V. Marańón T. Ojeda F. Clemente L. Redondo R. (2002). Seagull influence on soil properties, chenopod shrub distribution, and leaf nutrient status in semi-arid Mediterranean islands. *Oikos* 98 75–86. 10.1034/j.1600-0706.2002.980108.x

[B28] Gilham M. E. (1956). Ecology of the Pembrokeshire Islands. V. Manuring by the colonial seabirds and mammals, with a note on seed distribution by gulls. *J. Ecol.* 44 429–454. 10.2307/2256831

[B29] Grime J. P. (1979). “Competition and the struggle for existence,” in *Proceedings of the 20th British Ecological Society Symposium London, England: Population Dynamics*, eds Anderson R. M. Turner B. D. Taylor L. R. (Oxford: Blackwell Scientific Publications), 123–139.

[B30] Hill P. Farrar J. Roberts P. Farrell M. Grant H. Newsham K. (2011). Vascular plant success in a warming Antarctic may be due to efficient nitrogen acquisition. *Nat. Clim. Change* 1 50–53. 10.1038/nclimate1060

[B31] Hodkinson I. D. Coulson S. J. Webb N. R. (2003). Community assembly along proglacial chronosequences in the high Arctic: vegetation and soil development in north-west Svalbard. *J. Ecol.* 91 651–663. 10.1046/j.1365-2745.2003.00786.x

[B32] Hoek C. Mann D. Jahns H. M. (1995). *Algae: An Introduction to Phycology.* Cambridge: Cambridge university press.

[B33] Holm S. (1979). A simple sequentially rejective multiple test procedure. *Scand. J. Stat.* 6 65–70.

[B34] Hutchinson G. E. (1950). Survey of existing knowledge of biogeochemistry. 3. The biogeochemistry of vertebrate excretion. *Bull. Am. Mus. Nat. Hist.* 96 483–519.

[B35] Isaksen K. (1995). *Distribution of Seabirds at Sea in the Northern Barents Sea. Seabird Populations in the Northern Barents Sea. Source Data for the Impact Assessment of the Effects of Oil Drilling Activity. Norsk Polarinstitutt Medd 135*, (Tromsø: Norsk Polarinstitutt), 67–80.

[B36] Jakubas D. Zmudczyńska K. Wojczulanis-Jakubas K. Stempniewicz L. (2008). Faeces deposition and numbers of vertebrate herbivores in the vicinity of planktivorous and piscivorous seabird colonies in Hornsund, Spitsbergen. *Pol. Polar Res.* 29 45–58.

[B37] Kelly J. F. (2000). Stable isotopes of carbon and nitrogen in the study of avian and mammalian trophic ecology. *Can. J. Zool.* 78 1–27. 10.1139/z99-165

[B38] Komárek J. (2016). Review of the cyanobacterial genera implying planktic species after recent taxonomic revisions according to polyphasic methods: state as of 2014. *Hydrobiologia* 764 259–270. 10.1007/s10750-015-2242-0

[B39] Komárek J. Anagnostidis K. (1999). “Cyanoprokaryota 1. Teil Chroococcales,” in *Sußwasserflora von Mitteleuropa*, eds Ettl H. Gartner G. Heynig H. Mollenhauer D. (Gustav Fischer: Jena-Stuttgart-Lubeck-Ulm).

[B40] Kristinsson H. Zhurbenko M. Hansen E. S. (2010). *Panarctic Checklist of Lichens and Lichenicolous Fungi.* CAFF Technical Report No. 20 CAFF International Secretariat. Akureyri: CAFF International Secretariat.

[B41] Matuła J. Pietryka M. Richter D. Wojtuń B. (2007). Cyanoprokaryota and algae of Arctic terrestrial ecosystems in the Horsund area, Spitsbergen. *Pol. Polar Res.* 28 283–315.

[B42] Mulder C. P. H. Jones H. Kameda K. Palmborg C. Schmidt S. Ellis J. C. (2011). “Impacts of seabirds on plant and soil properties,” in *Seabird Islands: Ecology, Invasion and Restoration*, eds Mulder C. P. H. Anderson W. B. Towns D. R. Bellingham P. J. (New York, NY: Oxford University Press), 135–176.

[B43] Nakatsubo T. Fujiyoshi M. Yoshitake S. Koizumi H. Uchida M. (2010). Colonization of the polar willow Salix polaris on the early stage of succession after glacier retreat in the High Arctic, Ny-Ålesund, Svalbard. *Polar Res.* 29 385–390. 10.1111/j.1751-8369.2010.00170.x

[B44] Odasz A. M. (1994). Nitrate reductase activity in vegetation below an arctic bird cliff, Svalbard, Norway. *J. Veg. Sci.* 5 913–920. 10.2307/3236203 PMC7201890 32390712

[B45] Odum E. P. (1993). *Ecology and Our Endangered Life-Support Systems.* Sunderland, MA: Sinauer Associates.

[B46] Øvstedal D. Tønsberg T. Elvebakk A. (2009). The lichen flora of Svalbard. *Sommerfeltia* 33:3.

[B47] Pietryka M. Richter D. Matuła J. (2016). Cyanobacterial and algal diversity in the vicinity of two different seabird colonies in Spitsbergen. *Pol. Polar Res.* 37 269–288. 10.1515/popore-2016-0015

[B48] Polis G. A. Anderson W. A. Holt R. D. (1997). Toward an integration of landscape and food web ecology: the dynamics of spatially subsidized food webs. *Annu. Rev. Ecol. Syst.* 28 289–316. 10.1146/annurev.ecolsys.28.1.289

[B49] Putman R. (1994). *Community Ecology.* New York, NY: Chapman and Hall.

[B50] Richter D. Matuła J. Pietryka M. (2009). Cyanoprokaryota and algae of selected tundra habitats in the Horsund fjord area (West Spitsbergen). *Oceanol. Hydrobiol. Stud.* 38 65–70.

[B51] Rønning O. S. (1996). *The Flora of Svalbard.* Oslo: Norsk Polarinstitut.

[B52] Skrzypek G. Wojtuń B. Richter D. Jakubas D. Wojczulanis-Jakubas K. Samecka-Cymerman A. (2015). Diversification of nitrogen sources in various tundra vegetation types in the High Arctic. *PLoS ONE* 10:e0136536 10.1371/journal.pone.0136536 PMC4574312 26376204

[B53] Smith V. R. Froneman P. W. (2008). “Nutrient dynamics in the vicinity of the Prince Edward Islands,” in *The Prince Edward Islands. Land-Sea Interactions in a Changing Ecosystem*, eds Chown S. L. Froneman P. W. (Stellenbosch: SUN Press), 165–179.

[B54] StatSoft Inc (2010). *STATISTICA (Data Analysis Software System), version 9.1.*

[B55] Stempniewicz L. (1990). Biomass of dovekie excreta in the vicinity of a breeding colony. *Colon. Waterbirds* 13 62–66. 10.2307/1521421

[B56] Stempniewicz L. Błachowiak-Samołyk K. Wêsławski J. M. (2007). Impact of climate change on zooplankton communities, seabird populations and arctic terrestrial ecosystem – A scenario. *Deep Sea Res. II* 54 2934–2945. 10.1016/j.dsr2.2007.08.012

[B57] Stempniewicz L. Zwolicki A. Iliszko L. Zmudczyńska K. Wojtuń B. (2006). Impact of plankton- and fish-eating seabird colonies on the Arctic tundra ecosystem—a comparison. *J. Ornithol.* 147 257–258.

[B58] Stewart K. J. Coxson D. Siciliano S. D. (2011). Small-scale spatial patterns in N2-fixation and nutrient availability in an arctic hummock–hollow ecosystem. *Soil Biol. Biochem.* 43 133–140. 10.1016/j.soilbio.2010.09.023

[B59] ter Braak C. Smilauer P. (2012). *Canoco Reference Manual and User’s Guide: Software for Ordination.* Ithaca, NY: Microcomputer Power.

[B60] Theodose T. A. Bowman W. D. (1997). The influence of interspecific competition on the distribution of an alpine graminoid: evidence for the importance of plant competition in an extreme environment. *Oikos* 79 63–74.

[B61] Van der Wal R. Bardgett R. D. Harrison R. D. Stien A. (2004). Vertebrate herbivores and ecosystem control: cascading effects of faeces on tundra ecosystem. *Ecography* 27 242–252. 10.1111/j.0906-7590.2004.03688.x

[B62] Van der Wal R. Loonen M. J. (1998). Goose droppings as food for reindeer. *Can. J. Zool.* 76 1117–1122. 10.1139/z98-033

[B63] Vanderpuye A. W. Elvebakk A. Nilsen L. (2002). Plant communities along environmental gradients of high-arctic mires in Sassendalen, Svalbard. *J. Veg. Sci.* 13 875–884. 10.1111/j.1654-1103.2002.tb02117.x

[B64] Vidal E. Jouventin P. Frenot Y. (2003). Contribution of alien and indigenous species to plant community assemblages near penguin rookeries at Crozet archipelago. *Polar Biol.* 26 432–437.

[B65] Węgrzyn M. Wietrzyk P. (2015). Phytosociology of snowbed and exposed ridge vegetation of Svalbard. *Polar Biol.* 38 1905–1917. 10.1007/s00300-015-1751-7

[B66] Weydmann A. Carstensen J. Goszczko I. Dmoch K. Olszewska A. Kwaśniewski S. (2014). Shift towards the dominance of boreal species in the Arctic: inter-annual and spatial zooplankton variability in the West Spitsbergen Current. *Mar. Ecol. Progress Ser.* 501 41–52. 10.3354/meps10694

[B67] Wirth V. (2010). Ökologische Zeigerwerte von Flechten-erweiterte und aktualisierte Fassung. *Herzogia* 23 229–248. 10.13158/heia.23.2.2010.229

[B68] Wojciechowska A. Zwolicki A. Barcikowski A. Stempniewicz L. (2015). The structure of Cochlearia groenlandica population along the bird colony influence gradient (Hornsund, Spitsbergen). *Polar Biol.* 38 1919–1930. 10.1007/s00300-015-1755-3

[B69] Wojczulanis-Jakubas K. Jakubas D. Welcker J. Harding A. M. Karnovsky N. J. Kidawa D. (2011). Body size variation of a high-Arctic seabird: the dovekie (Alle alle). *Polar Biol.* 34 847–854. 10.1007/s00300-010-0941-6

[B70] Zawierucha K. Zmudczyńska-Skarbek K. Kaczmarek Ł Wojczulanis-Jakubas K. (2015). The influence of a seabird colony on abundance and species composition of water bears (Tardigrada) in Hornsund (Spitsbergen, Arctic). *Polar Biol.* 34 713–723.

[B71] Zielke M. Solenheim B. Spjelkaavik S. Olsen R. (2005). Nitrogen Fixation in the High Arctic: role of vegetation and environmental conditions. *Arct. Antarct. Alp. Res.* 37 372–378. 10.1657/1523-0430(2005)037[0372:NFITHA]2.0.CO;2

[B72] Zmudczyńska K. Olejniczak I. Zwolicki A. Iliszko L. Convey P. Stempniewicz L. (2012). Influence of allochtonous nutrients delivered by colonial seabirds on soil collembolan communities on Spitsbergen. *Polar Biol.* 35 1233–1245. 10.1007/s00300-012-1169-4

[B73] Zmudczyńska-Skarbek K. Barcikowski M. Zwolicki A. Iliszko L. Stempniewicz L. (2013). Variability of polar scurvygrass Cochlearia groenlandica individual traits along a seabird influenced gradient across Spitsbergen tundra. *Polar Biol.* 36 1659–1669. 10.1007/s00300-013-1385-6

[B74] Zmudczyńska-Skarbek K. Zwolicki A. Convey P. Barcikowski M. Stempniewicz L. (2015). Is ornithogenic fertilisation important for collembolan communities in Arctic terrestrial ecosystems? *Polar Res.* 34:25629.

[B75] Zwolicki A. Barcikowski M. Barcikowski A. Cymerski M. Stempniewicz L. Convey P. (2015). Seabird colony effects on soil properties and vegetation zonation patterns on King George Island, Maritime Antarctic. *Polar Biol.* 38 1645–1655. 10.1007/s00300-015-1730-z

[B76] Zwolicki A. Zmudczyńska-Skarbek K. Richard P. Stempniewicz L. (2016). Importance of marine-derived nutrients supplied by planktivorous seabirds to High Arctic Tundra Plant Communities. *PLoS ONE* 11:e0154950 10.1371/journal.pone.0154950 PMC4858296 27149113

[B77] Zwolicki A. Zmudczyńska-Skarbek K. M. Iliszko L. Stempniewicz L. (2013). Guano deposition and nutrient enrichment in the vicinity of planktivorous and piscivorous seabird colonies in Spitsbergen. *Polar Biol.* 36 363–372. 10.1007/s00300-012-1265-5

