# Supplementary material for: Differential Responses of Arctic Vegetation to Nutrient Enrichment by Plankton- and Fish-Eating Colonial Seabirds in Spitsbergen
Source: Front Plant Sci. 2016 Dec 27;7:1959. doi: 10.3389/fpls.2016.01959 (PMC5187377; doi:10.3389/fpls.2016.01959)
Supplement: Supplementary file 1 [file Table_1.DOCX]

Supplementary Material

Differential responses of tundra vegetation to nutrient enrichment by plankton- and fish-eating colonial seabirds in Spitsbergen

**Adrian Zwolicki^1*^, Katarzyna Zmudczyńska-Skarbek^1^, Jan Matuła^2^, Bronisław Wojtuń^3^, Lech Stempniewicz^1^**

***Correspondence:** Adrian Zwolicki, Dept. of Vertebrate Ecology and Zoology, University of Gdańsk, Wita Stwosza 59, 80-308 Gdańsk, Poland

e-mail: adrian.zwolicki@ug.edu.pl,

Table S1. Characteristics of the five distinguished LINKTREE groups with average percentage ground cover: (AC) and similarity within each group (AS) based on SIMPER analysis. Taxa listed according to the average total coverage. In each group, AC and AS values of two dominant species are marked with boxes.

|  |  | |  | | **LinkTree groups** | |  | |  | |
| --- | --- | --- | --- | --- | --- | --- | --- | --- | --- | --- |
|  | **Group G1** | | **Group G2** | | **Group G3** | | **Group G4** | | **Group G5** | |
| Community | *C. groenlandica-* *P. alpina* | | *D. alpina- C. arcticum* | | *S. uncinata- S. stramineum* | | *S. uncinata- S. oppositifolia* | | *S. uncinata S. polaris* | |
| N plots | 7 | | 6 | | 4 | | 7 | | 18 | |
| N vascular plants | 9 | | 13 | | 9 | | 12 | | 22 | |
| N mosses | 11 | | 17 | | 18 | | 24 | | 31 | |
| N algae | 17 | | 18 | | 13 | | 10 | | 30 | |
| N lichens | 0 | | 3 | | 9 | | 14 | | 40 | |
| N total taxa | 44 | | 57 | | 53 | | 67 | | 141 | |
| Average similarity | 38.96 | | 25.45 | | 15.76 | | 22.17 | | 44.76 | |
| **Taxa** | **AC** | **AS** | **AC** | **AS** | **AC** | **AS** | **AC** | **AS** | **AC** | **AS** |
| *\* |  |  |  |  |  |  |  |  |  |  |
| *Sanionia uncinata* | 0.01 | 0 | 17.83 | 4.35 | 37.04 | 8.9 | 26.59 | 11.43 | 38.6 | 34.65 |
| *Cerastim arcticum* | 12.14 | 2.15 | 24.33 | 8.57 | 20.6 | 3.49 | 0.91 | 0.28 | 0.09 | 0.03 |
| *Cochlearia groenladica* | 42.14 | 28.62 | 1.03 | 0.49 | 0.6 | 0.08 | 0.06 | 0.07 | 0.03 | 0.01 |
| *Saxifraga oppositifolia* | 0 | 0 | 0 | 0 | 4.8 | 0 | 21.43 | 7.05 | 7.76 | 5.15 |
| *Deschampsia alpina* | 0 | 0 | 33.33 | 7.68 | 0 | 0 | 0.19 | 0.07 | 0.07 | 0.01 |
| *Poa alpina* | 16.01 | 6.55 | 6.72 | 1.82 | 6.4 | 0.85 | 0 | 0 | 0 | 0 |
| *Salix polaris* | 0 | 0 | 7.5 | 0.36 | 4 | 0 | 0 | 0 | 10.25 | 4.11 |
| *Straminergon stramineum* | 0 | 0 | 0 | 0 | 20.8 | 1.28 | 0 | 0 | 0.4 | 0 |
| *Festuca rubra* | 7.16 | 0.36 | 6.67 | 0 | 0 | 0 | 1.71 | 0.1 | 0.01 | 0 |
| *Saxifraga caespitosa* | 10.01 | 0.7 | 0.52 | 0 | 3.62 | 0.01 | 0.49 | 0.2 | 0.36 | 0.13 |
| *Cyrtomnium hymenophylloides* | 1.44 | 0 | 5.83 | 0 | 0 | 0 | 0.01 | 0 | 0 | 0 |
| *Ceratodon purpureus* | 0 | 0 | 0.85 | 0.2 | 0.62 | 0.01 | 4.19 | 0.93 | 0.02 | 0.01 |
| *Saxifraga hyperborea* | 0.01 | 0 | 4.83 | 0.29 | 0.64 | 0.13 | 0.03 | 0.01 | 0.02 | 0 |
| *Bryum* sp*.* | 2.33 | 0.03 | 0.35 | 0.01 | 0.04 | 0.01 | 2.03 | 0.5 | 0.2 | 0.05 |
| *Plagiomnium elipticum* | 0.01 | 0 | 0 | 0 | 4 | 0.41 | 0.87 | 0.01 | 0 | 0 |
| *Tetraplodon mnioides* | 0 | 0 | 4 | 1.47 | 0.82 | 0.2 | 0 | 0 | 0 | 0 |
| *Syntrichia ruralis* | 0.01 | 0 | 0.57 | 0.03 | 0.4 | 0 | 2.3 | 0.43 | 0.66 | 0.1 |
| *Philonotis tomentella* | 0 | 0 | 3.33 | 0 | 0 | 0 | 0 | 0 | 0 | 0 |
| *Chrysosplenium tetrandum* | 0 | 0 | 0.52 | 0.01 | 2.6 | 0.24 | 0 | 0 | 0.01 | 0 |
| *Bryum pallescens* | 2.57 | 0.25 | 0.18 | 0.01 | 0 | 0 | 0 | 0 | 0 | 0 |
| *Aulacomium palustre* | 0 | 0 | 0 | 0 | 1.06 | 0.05 | 1.43 | 0 | 0.2 | 0 |
| *Brachytecium turgidum* | 0.01 | 0 | 0.4 | 0.12 | 0.22 | 0.01 | 1.47 | 0.85 | 0.21 | 0.03 |
| *Saxifraga cernua* | 1.46 | 0.3 | 0.05 | 0.01 | 0 | 0 | 0.2 | 0.1 | 0.03 | 0.02 |
| *Timmia austriaca* | 0 | 0 | 0.83 | 0 | 0 | 0 | 0.89 | 0.02 | 0 | 0 |
| *Rhacomitrium canescens* | 0 | 0 | 0 | 0 | 0 | 0 | 0 | 0 | 1.36 | 0.1 |
| *Ditrichum flexicaule* | 0 | 0 | 0 | 0 | 0 | 0 | 0.87 | 0.01 | 0 | 0 |
| *Oncophorus wahlenbergii* | 0 | 0 | 0 | 0 | 0 | 0 | 0.01 | 0 | 0.83 | 0.19 |
| *Ptilidium ciliare* | 0 | 0 | 0 | 0 | 0.6 | 0 | 0 | 0 | 0.09 | 0.02 |
| *Tomenthypnum nitens* | 0 | 0 | 0 | 0 | 0 | 0 | 0.57 | 0 | 0.01 | 0 |
| *Bryum pseudotriquetrum* | 0 | 0 | 0.33 | 0 | 0.2 | 0 | 0.03 | 0 | 0.01 | 0 |
| *Climacium dendroides* | 0.01 | 0 | 0.5 | 0 | 0 | 0 | 0 | 0 | 0 | 0 |
| *Polytrichastrum alpinum* | 0 | 0 | 0 | 0 | 0.22 | 0.01 | 0.01 | 0 | 0.19 | 0.06 |
| *Luzula confusa* | 0 | 0 | 0 | 0 | 0.4 | 0.08 | 0 | 0 | 0 | 0 |
| *Phippsia algida* | 0 | 0 | 0.33 | 0 | 0 | 0 | 0 | 0 | 0.02 | 0.01 |
| *Distichum capillaceum* | 0 | 0 | 0.02 | 0 | 0 | 0 | 0.04 | 0.02 | 0.28 | 0.01 |
| *Warnstorfia* sp*.* | 0 | 0 | 0.05 | 0.02 | 0.2 | 0 | 0 | 0 | 0 | 0 |
| *Silene acaulis* | 0 | 0 | 0.02 | 0 | 0 | 0 | 0 | 0 | 0.2 | 0.02 |
| *Pohlia cruda* | 0 | 0 | 0 | 0 | 0.2 | 0 | 0 | 0 | 0 | 0 |
| *Saxifraga nivalis* | 0 | 0 | 0.02 | 0 | 0 | 0 | 0.17 | 0.02 | 0 | 0 |
| *Cerastium regelii* | 0 | 0 | 0 | 0 | 0 | 0 | 0.17 | 0.04 | 0.01 | 0 |
| *Aulacomium turgidum* | 0 | 0 | 0 | 0 | 0 | 0 | 0.14 | 0 | 0 | 0 |
| *Draba alpina* | 0 | 0 | 0.02 | 0 | 0.02 | 0 | 0.01 | 0 | 0.01 | 0 |
| *Lophoziaceae* | 0 | 0 | 0 | 0 | 0 | 0 | 0.04 | 0.02 | 0.02 | 0 |
| *Hypnum* sp*.* | 0 | 0 | 0.02 | 0 | 0 | 0 | 0.03 | 0.01 | 0.01 | 0 |
| *Polygonum viviparum* | 0 | 0 | 0 | 0 | 0 | 0 | 0 | 0 | 0.05 | 0.03 |
| *Sagina nivalis* | 0 | 0 | 0 | 0 | 0 | 0 | 0.01 | 0 | 0.04 | 0.02 |
| *Brachythecium albicans* | 0.01 | 0 | 0 | 0 | 0 | 0 | 0.03 | 0.01 | 0 | 0 |
| *Pohlia nutans* | 0 | 0 | 0 | 0 | 0.02 | 0 | 0.01 | 0 | 0.01 | 0 |
| *Equisetum boreale* | 0 | 0 | 0 | 0 | 0 | 0 | 0 | 0 | 0.03 | 0.01 |
| *Hypnum revolutum* | 0 | 0 | 0 | 0 | 0 | 0 | 0.01 | 0 | 0.02 | 0 |
| *Bryum wrightii* | 0 | 0 | 0.02 | 0 | 0 | 0 | 0 | 0 | 0 | 0 |
| *Campylium* sp*.* | 0 | 0 | 0 | 0 | 0 | 0 | 0.01 | 0 | 0.01 | 0 |
| *Dicranum fuscescens* | 0 | 0 | 0 | 0 | 0.02 | 0 | 0 | 0 | 0 | 0 |
| *Mnium* sp*.* | 0 | 0 | 0 | 0 | 0.02 | 0 | 0 | 0 | 0 | 0 |
| *Pohlia* sp*.* | 0.01 | 0 | 0 | 0 | 0 | 0 | 0 | 0 | 0.01 | 0 |
| *Pseudoleskeella catenulata* | 0 | 0 | 0 | 0 | 0 | 0 | 0.01 | 0 | 0.01 | 0 |
| *Blepharostoma trichophyllum* | 0 | 0 | 0 | 0 | 0 | 0 | 0 | 0 | 0.01 | 0 |
| *Blindia* sp*.* | 0 | 0 | 0 | 0 | 0 | 0 | 0 | 0 | 0.01 | 0 |
| *Brachythecium* sp*.* | 0 | 0 | 0 | 0 | 0 | 0 | 0 | 0 | 0.01 | 0 |
| *Calliergon richardsonii* | 0 | 0 | 0 | 0 | 0 | 0 | 0 | 0 | 0.01 | 0 |
| *Campylium polygamum* | 0 | 0 | 0 | 0 | 0 | 0 | 0 | 0 | 0.01 | 0 |
| *Cephaloziella arctica* | 0 | 0 | 0 | 0 | 0 | 0 | 0 | 0 | 0.01 | 0 |
| *Deschampsia caespitosa* | 0 | 0 | 0 | 0 | 0 | 0 | 0 | 0 | 0.01 | 0 |
| *Dicranum* sp*.* | 0 | 0 | 0 | 0 | 0 | 0 | 0 | 0 | 0.01 | 0 |
| *Draba* sp*.* | 0 | 0 | 0 | 0 | 0 | 0 | 0 | 0 | 0.01 | 0 |
| *Drepanocladus* sp*.* | 0 | 0 | 0 | 0 | 0 | 0 | 0 | 0 | 0.01 | 0 |
| *Haplodon wormskjoldii* | 0.01 | 0 | 0 | 0 | 0 | 0 | 0 | 0 | 0 | 0 |
| *Juncus biglumis* | 0 | 0 | 0 | 0 | 0 | 0 | 0 | 0 | 0.01 | 0 |
| *Philonotis* sp*.* | 0 | 0 | 0 | 0 | 0 | 0 | 0 | 0 | 0.01 | 0 |
| *Ranunculus sulfurea* | 0.01 | 0 | 0 | 0 | 0 | 0 | 0 | 0 | 0 | 0 |
| *Sagina* sp*.* | 0 | 0 | 0 | 0 | 0 | 0 | 0 | 0 | 0.01 | 0 |
| *Salix reticulata* | 0 | 0 | 0 | 0 | 0 | 0 | 0 | 0 | 0.01 | 0 |
| *Saxifraga rivularis* | 0.01 | 0 | 0 | 0 | 0 | 0 | 0 | 0 | 0 | 0 |
| *Saxifraga tenuis* | 0 | 0 | 0 | 0 | 0 | 0 | 0 | 0 | 0.01 | 0 |
| *Tortula* sp*.* | 0 | 0 | 0 | 0 | 0 | 0 | 0.01 | 0 | 0 | 0 |
