# Supplementary material for: Differential Responses of Arctic Vegetation to Nutrient Enrichment by Plankton- and Fish-Eating Colonial Seabirds in Spitsbergen
Source: Front Plant Sci. 2016 Dec 27;7:1959. doi: 10.3389/fpls.2016.01959 (PMC5187377; doi:10.3389/fpls.2016.01959)
Supplement: Supplementary file 2 [file Table_2.DOCX]

***Supplementary Material***

**Differential responses of tundra vegetation to nutrient enrichment by plankton- and fish-eating colonial seabirds in Spitsbergen**

**Adrian Zwolicki^1*^, Katarzyna Zmudczyńska-Skarbek^1^, Jan Matuła^2^, Bronisław Wojtuń^3^, Lech Stempniewicz^1^**

***Correspondence:** Adrian Zwolicki, Dept. of Vertebrate Ecology and Zoology, University of Gdańsk, Wita Stwosza 59, 80-308 Gdańsk, Poland

e-mail: adrian.zwolicki@ug.edu.pl,

Table S3. Lichen taxa occurrences (+) within five groups distinguished by LINKTREE analysis.

| **Taxa** | **G1** | **G2** | **G3** | **G4** | **G5** |
| --- | --- | --- | --- | --- | --- |
| *Bacidia bagliettoana* |  |  |  |  | + |
| *Biatora subduplex* |  |  |  |  | + |
| *Bilimbia microcarpa* |  |  |  |  | + |
| *Bilimbia sabuletorum* |  |  |  | + | + |
| *Buellia insignis* |  |  |  |  | + |
| *Caloplaca* sp*.* |  |  |  | + |  |
| *Caloplaca stilicidiorum* |  |  |  | + | + |
| *Caloplaca tetraspora* |  |  |  |  | + |
| *Caloplaca tiroliensis* |  |  |  |  | + |
| *Caloplaca tornoënsis* |  |  |  |  | + |
| *Cetrariella delisei* |  |  | + | + | + |
| *Cladonia arbuscula* subsp*. mitis* |  |  | + |  | + |
| *Cladonia* cf*. borealis* |  |  | + |  |  |
| *Cladonia* cf*. ecomcyna* |  |  |  |  | + |
| *Cladonia* cf*. fimbriata* |  |  | + |  |  |
| *Cladonia pyxidata* s.l*.* |  |  | + |  | + |
| *Cladonia* sp. |  |  | + | + | + |
| *Collema ceraniscum* |  |  |  | + | + |
| *Collema* sp. |  |  |  |  | + |
| *Flavocetraria cucullata* |  |  |  | + |  |
| *Lecania subfuscula* |  |  |  | + | + |
| *Lecanora dispersa* s.l*.* |  |  |  |  | + |
| *Lecanora hagenii* |  |  |  |  | + |
| *Lecanora semipallida* |  |  |  |  | + |
| *Lecanora zosterae* |  |  |  |  | + |
| *Lecidea* cf. *alpestris* |  |  |  |  | + |
| *Lecidella* sp. |  |  |  |  | + |
| *Leptogium* sp. |  |  |  | + | + |
| *Megaspora verrucosa* |  |  |  |  | + |
| *Micarea* sp. |  |  |  |  | + |
| *Mycobilimbia berengeriana* |  |  |  |  | + |
| *Mycobilimbia hypnorum* |  |  |  |  | + |
| *Mycobilimbia* sp. |  |  |  |  | + |
| *Ochrolechia frigida* |  |  | + | + | + |
| *Peltigera canina* |  |  |  | + |  |
| *Peltigera rufescens* |  |  |  | + | + |
| *Pertusaria* sp. |  |  |  |  | + |
| *Physcia caesia* |  | + |  |  |  |
| *Physcia dubia* |  | + |  |  |  |
| *Physconia muscigena* |  |  | + |  |  |
| *Polyblastia sendtneri* |  |  |  |  | + |
| *Psoroma hypnorum* |  |  |  |  | + |
| *Rinodina mniaraea* |  |  |  |  | + |
| *Rinodina septentrionalis* |  |  |  |  | + |
| *Santessoniella arctophila* |  |  |  |  | + |
| *Solorina bispora* |  |  |  |  | + |
| *Solorina spongiosa* |  |  |  | + |  |
| *Stereocaulon* sp. |  |  | + |  | + |
| *Thamnolia vermicularis* |  |  |  | + |  |
| *Xanthoria candelaria* |  | + |  |  |  |
