# Supplementary material for: Differential Responses of Arctic Vegetation to Nutrient Enrichment by Plankton- and Fish-Eating Colonial Seabirds in Spitsbergen
Source: Front Plant Sci. 2016 Dec 27;7:1959. doi: 10.3389/fpls.2016.01959 (PMC5187377; doi:10.3389/fpls.2016.01959)
Supplement: Supplementary file 3 [file Table_3.DOCX]

***Supplementary Material***

**Differential responses of tundra vegetation to nutrient enrichment by plankton- and fish-eating colonial seabirds in Spitsbergen**

**Adrian Zwolicki^1*^, Katarzyna Zmudczyńska-Skarbek^1^, Jan Matuła^2^, Bronisław Wojtuń^3^, Lech Stempniewicz^1^**

***Correspondence:** Adrian Zwolicki, Dept. of Vertebrate Ecology and Zoology, University of Gdańsk, Wita Stwosza 59, 80-308 Gdańsk, Poland

e-mail: adrian.zwolicki@ug.edu.pl,

Table S2. Occurrences of algae taxa (+) within five groups distinguished by LINKTREE analysis.

| **Taxa** | **G1** | **G2** | **G3** | **G4** | **G5** |
| --- | --- | --- | --- | --- | --- |
| *Aphanothece caldariorum* |  |  |  |  | + |
| *Aphanothece saxicola* |  |  |  |  | + |
| *Aphanothece* sp. |  |  | + |  | + |
| *Aphanothece stagnina* |  |  |  |  | + |
| *Borodinellopsis texensis* | + |  |  | + |  |
| *Calothrix gypsophila* |  |  |  |  | + |
| *Calothrix* sp. |  |  |  |  | + |
| *Chlamydomonas nivalis* |  | + | + |  |  |
| *Haematococcus pluvialis* | + | + |  | + |  |
| *Chondrocystis* cf. *dermochroa* |  |  |  |  | + |
| *Chroococcus* sp. | + | + |  |  |  |
| *Chroococcus turgidus* |  | + | + |  | + |
| *Cosmarium cucurbita* |  |  |  |  | + |
| *Excentrosphaera viridis* | + |  |  | + |  |
| Cf*. Fischeriella* sp*.* |  |  |  |  | + |
| *Gloeocapsa compacta* |  |  |  |  | + |
| *Gloeocapsa keutzingiana* |  |  |  |  | + |
| *Gloeocapsopsis (Gloeocapsa). magma* |  |  |  |  | + |
| *Gloeocapsa sanguinea* |  |  |  |  | + |
| *Gloeocapsa* sp. | + |  | + | + | + |
| *Homeothrix* sp. |  |  |  |  | + |
| *Klebsormidium* sp. | + |  |  |  |  |
| *Leptolyngbya* cf. *foveolarum* |  | + |  |  |  |
| *Leptolyngbya* cf. *valderiana* |  |  |  |  | + |
| *Leptolyngbya* cf. *fragilis* |  | + | + |  |  |
| *Leptolyngbya* sp. | + |  |  |  |  |
| *Limnothrix* cf. *radekei* |  |  |  |  | + |
| *Limnothrix* sp. |  |  |  |  | + |
| *Merismopedia punctata* |  |  |  |  | + |
| *Merismopedia* sp. | + | + |  |  |  |
| *Merismopedia* cf. *tenuissima* | + | + | + |  |  |
| *Microcoleus vaginatus* |  |  |  |  | + |
| *Nostoc commune* |  |  |  | + | + |
| *Nostoc kihlmanii* |  |  |  |  | + |
| *Nostoc minutissima* |  |  |  | + |  |
| *Nostoc paludosum* | + |  |  | + | + |
| *Nostoc punctiforme* | + |  |  | + | + |
| *Nostoc* sp. |  |  |  |  | + |
| *Oscillatoria tenuis* |  |  | + |  |  |
| *Phormidium autumnale* | + | + | + |  |  |
| *Phormidium* cf. *uncinatum* | + |  |  |  |  |
| *Phormidium favosum* |  |  | + |  |  |
| *Prasiola crispa* | + | + | + |  |  |
| *Pseudanabaena sp.* | + | + | + |  |  |
| *Schizothrix* cf*. fragilis* |  |  | + |  |  |
| *Schizothrix* sp. |  | + |  |  | + |
| *Scotiellopsis* sp. |  | + |  |  |  |
| *Scotiellopsis terrestris* |  |  | + |  |  |
| *Scytonema crustaceum* |  |  |  | + | + |
| *Tribonema* sp. |  | + |  |  |  |
| *Trichomus variabilis* |  |  |  | + |  |
| *Trochiscia granulata* |  | + |  |  |  |
| *Trochiscia prescottii* |  | + |  |  |  |
| *Trochiscia* sp. |  | + |  |  |  |
| *Ulothrix subtilis* |  | + |  |  |  |
| *Ulothrix warabilis* | + |  |  |  |  |
| *Woronichinia compacta* | + |  |  |  |  |
