# Supplementary material for: Differential Responses of Arctic Vegetation to Nutrient Enrichment by Plankton- and Fish-Eating Colonial Seabirds in Spitsbergen
Source: Front Plant Sci. 2016 Dec 27;7:1959. doi: 10.3389/fpls.2016.01959 (PMC5187377; doi:10.3389/fpls.2016.01959)
Supplement: Supplementary file 4 [file Table_4.DOCX]

***Supplementary Material***

**Differential responses of tundra vegetation to nutrient enrichment by plankton- and fish-eating colonial seabirds in Spitsbergen**

**Adrian Zwolicki^1*^, Katarzyna Zmudczyńska-Skarbek^1^, Jan Matuła^2^, Bronisław Wojtuń^3^, Lech Stempniewicz^1^**

***Correspondence:** Adrian Zwolicki, Dept. of Vertebrate Ecology and Zoology, University of Gdańsk, Wita Stwosza 59, 80-308 Gdańsk, Poland

e-mail: adrian.zwolicki@ug.edu.pl,

Table S4. Pairwise comparison matrix (Tuckey, RIR test) of *δ*^15^N (‰) and total nitrogen content (%) between five distinguished LINKTREE groups (G1-G5). Significance levels: *** - *p* < 0.001, ** - *p* < 0.01., * - *p* < 0.05, ns – non-significant. S – soil, Vp – vascular plants, M – mosses.

|  |  | **G1** | | | **G2** | | | **G3** | | | **G4** | | |
| --- | --- | --- | --- | --- | --- | --- | --- | --- | --- | --- | --- | --- | --- |
|  |  | **S** | **Vp** | **M** | **S** | **Vp** | **M** | **S** | **Vp** | **M** | **S** | **Vp** | **M** |
| **G2** | ***δ*^15^N (‰)** | *** | ns | ns | - | - | - | - | - | - | - | - | - |
|  | **N (%)** | ns | ns | ns | - | - | - | - | - | - | - | - | - |
| **G3** | ***δ*^15^N (‰)** | ** | ns | ns | ns | ns | ns | - | - | - | - | - | - |
|  | **N (%)** | ns | ns |  | ns | ns | ns | - | - | - | - | - | - |
| **G4** | ***δ*^15^N (‰)** | *** | ** | ** | ** | ns | ns | ns | ns | ns | - | - | - |
|  | **N (%)** | ns | ns |  | ns | ns | ** | ** | ns | ns | - | - | - |
| **G5** | ***δ*^15^N (‰)** | *** | *** | *** | *** | ** | ns | ** | ns | ns | ns | ns | ns |
|  | **N (%)** | *** | ** | ns | *** | ns | ** | ** | ns | * | ns | ns | ns |
