# Supplementary material for: Differential Responses of Arctic Vegetation to Nutrient Enrichment by Plankton- and Fish-Eating Colonial Seabirds in Spitsbergen
Source: Front Plant Sci. 2016 Dec 27;7:1959. doi: 10.3389/fpls.2016.01959 (PMC5187377; doi:10.3389/fpls.2016.01959)
Supplement: Supplementary file 5 [file Image_1.PDF]

Supplementary Material

Differential responses of tundra vegetation to nutrient enrichment by plankton- and fish-eating colonial seabirds in Spitsbergen

Adrian Zwolicki[1*], Katarzyna Zmudczyńska-Skarbek[1], Jan Matuła[2], Bronisław Wojtuń[3], Lech Stempniewicz[1]

***Correspondence:** Adrian Zwolicki, Dept. of Vertebrate Ecology and Zoology, University of Gdańsk, Wita Stwosza 59, 80-308 Gdańsk, Poland, e-mail: adrian.zwolicki@ug.edu.pl,

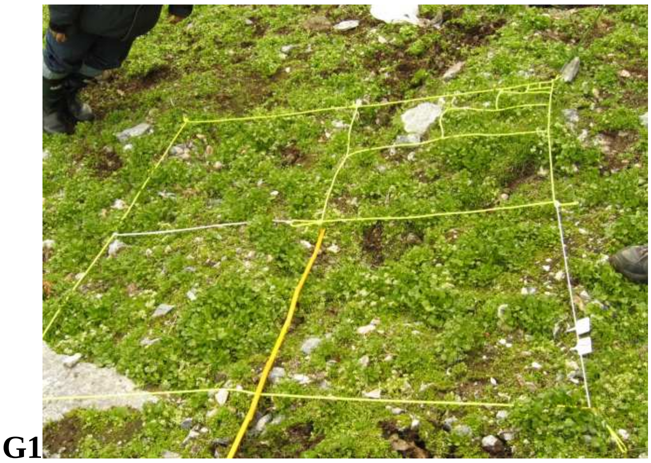

G1

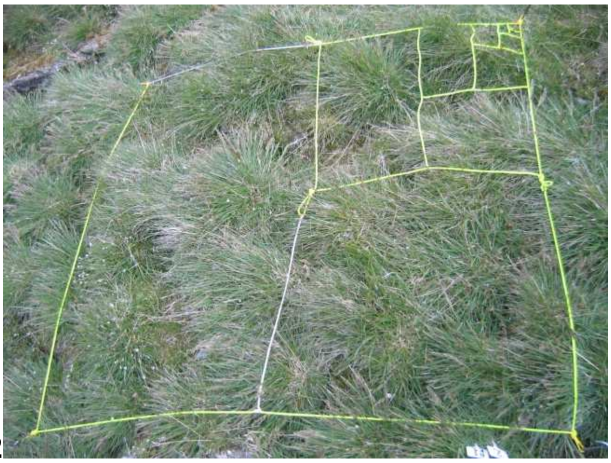

G2

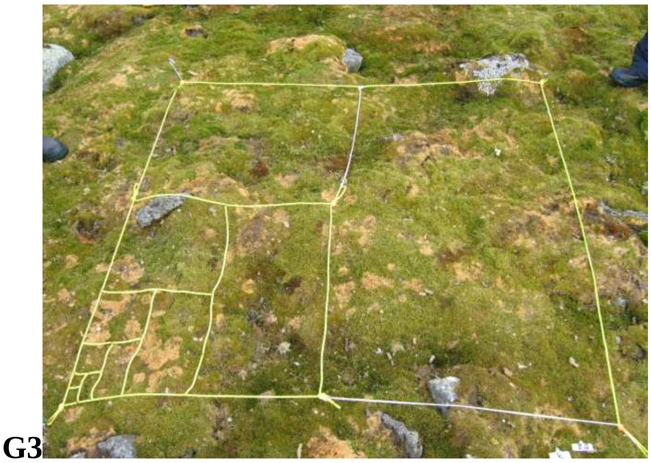

G3

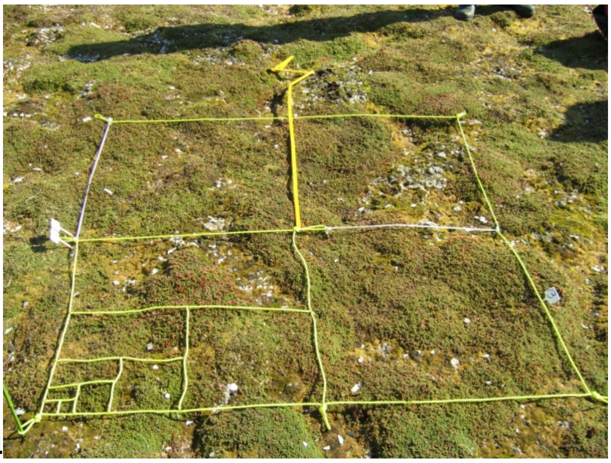

G4

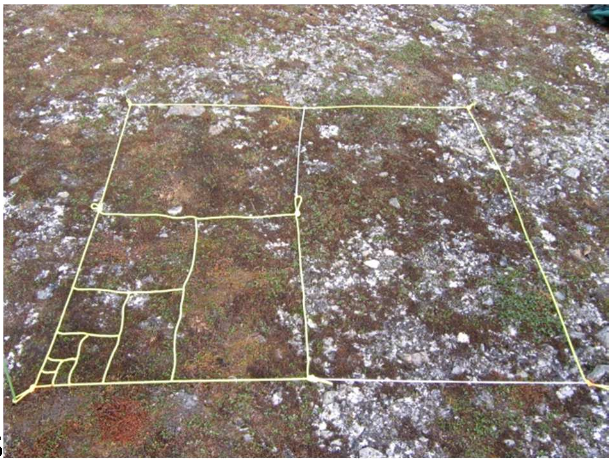

G5

Figure S1. Sample plots pictures of typical representatives of communities distinguished by LINKTREE (Photography by A. Zwolicki).
